# Conformational switching of the pseudokinase domain promotes human MLKL tetramerization and cell death by necroptosis

**DOI:** 10.1038/s41467-018-04714-7

**Published:** 2018-06-21

**Authors:** Emma J. Petrie, Jarrod J. Sandow, Annette V. Jacobsen, Brian J. Smith, Michael D. W. Griffin, Isabelle S. Lucet, Weiwen Dai, Samuel N. Young, Maria C. Tanzer, Ahmad Wardak, Lung-Yu Liang, Angus D. Cowan, Joanne M. Hildebrand, Wilhelmus J. A. Kersten, Guillaume Lessene, John Silke, Peter E. Czabotar, Andrew I. Webb, James M. Murphy

**Affiliations:** 1 The Walter & Eliza Hall Institute of Medical Research, Parkville, VIC Australia; 2 Department of Medical Biology, The University of Melbourne, Parkville, VIC Australia; 3 Department of Biochemistry & Molecular Biology, LaTrobe University, Bundoora, VIC Australia; 4 Department of Biochemistry & Molecular Biology, Bio21 Molecular Science and Biotechnology Institute, The University of Melbourne, The Bio21 Institute, Parkville, VIC Australia; 5 Department of Pharmacology and Therapeutics, The University of Melbourne, Parkville, VIC Australia; 6 Present Address: Department of Proteomics and Signal Transduction, Max Planck Institute of Biochemistry, Martinsried, Bavaria Germany

## Abstract

Necroptotic cell death is mediated by the most terminal known effector of the pathway, MLKL. Precisely how phosphorylation of the MLKL pseudokinase domain activation loop by the upstream kinase, RIPK3, induces unmasking of the N-terminal executioner four-helix bundle (4HB) domain of MLKL, higher-order assemblies, and permeabilization of plasma membranes remains poorly understood. Here, we reveal the existence of a basal monomeric MLKL conformer present in human cells prior to exposure to a necroptotic stimulus. Following activation, toggling within the MLKL pseudokinase domain promotes 4HB domain disengagement from the pseudokinase domain αC helix and pseudocatalytic loop, to enable formation of a necroptosis-inducing tetramer. In contrast to mouse MLKL, substitution of RIPK3 substrate sites in the human MLKL pseudokinase domain completely abrogated necroptotic signaling. Therefore, while the pseudokinase domains of mouse and human MLKL function as molecular switches to control MLKL activation, the underlying mechanism differs between species.

## Abstract (web summary)

RIPK3-mediated phosphorylation of the mixed lineage kinase domain-like (MLKL) pseudokinase is thought to be the trigger for MLKL activation during necroptotic signaling. Here the authors provide evidence that the transition of human MLKL from a monomeric state to a tetramer is essential for necroptosis signalling.

## Introduction

Necroptosis is a regulated cell death mechanism in which the plasma membrane is compromised, allowing escape of the cell’s contents and instigation of an inflammatory response. In contrast to apoptosis, necroptosis is a caspase-independent cell death pathway, which is executed by the terminal protein, mixed lineage kinase domain-like (MLKL), following activation by the conventional protein kinase, receptor interacting protein kinase (RIPK)-3^1–3^. RIPK3 hetero-oligomerizes with another protein kinase, RIPK1, to form the “necrosome”: a high molecular weight signaling platform that has been variously reported to activate MLKL via stable recruitment^2,3^ or by a transient enzyme–substrate interaction^1^ with RIPK3. RIPK3-mediated phosphorylation of the activation loop residues, mouse S345^1,4,5^ or human T357/S358^2^, in the MLKL pseudokinase domain (PsKD) is widely thought to be the trigger for MLKL activation. MLKL phosphorylation promotes oligomerization, translocation to the inner plasma membrane, and ensuing necroptotic cell death by membrane permeabilization^6–12^, although the precise molecular details of this event are the subject of ongoing debate.

MLKL comprises an N-terminal four-helix bundle (4HB) domain connected to a C-terminal PsKD via a two-helix linker, which we termed the brace helices^1^. The N-terminal 4HB domain was shown by us and others using cellular and biochemical assays to be responsible for lipid engagement and membrane permeabilization^7,10,12,13^. The PsKD is so-named because it has a conserved kinase-like fold^1,5,14^; however, it lacks the residues that are necessary for catalytic activity in canonical protein kinases. As a result, we hypothesized that the PsKD functions as a molecular switch, where phosphorylation of the MLKL PsKD activation loop by RIPK3 leads to a conformation change and relief of an inhibitory protein–protein interaction with the executioner 4HB domain^1,11,15^. Despite lacking catalytic activity, MLKL has retained the ability to bind ATP^1,14,16^, although the role of nucleotide binding in modulating the molecular switch and regulating MLKL’s necroptotic function are currently unclear.

How the PsKD might suppress the executioner function of the 4HB domain, and the nature of the conformational change, if any, that occurs upon MLKL activation, remain unknown. Much of our understanding of mechanism extends from the structure of full-length mouse MLKL^1^, where the 4HB domain is solvent exposed and would thus be available to participate in necroptotic killing. In contrast, no structure of full-length human MLKL has been reported to date, and while recombinant mouse MLKL forms trimers in solution^11^, the stoichiometry of human MLKL oligomers has been a matter of debate. Intriguingly, in contrast to mouse MLKL, human MLKL 4HB domain expression does not induce cell death in mouse or human cells in the absence of forced oligomerization^7,17^. Additionally, in contrast to recombinant mouse MLKL 4HB+brace and full-length human MLKL, human MLKL 4HB+brace exhibited modest activity in liposome permeabilization assays in vitro^7^, implying a crucial role for the PsKD in a necroptotic human MLKL assembly.

Using biophysics, mass spectrometry (MS), and cellular assays, we reveal a role for the PsKD in directing the transition of human MLKL from a basal monomeric state to a pro-necroptotic tetramer. Wild-type hMLKL assembled into tetramers in vitro, robustly permeabilized liposomes, and reconstituted necroptotic signaling in *MLKL*^*−/−*^ U937 cells. In contrast, hMLKL PsKD mutants, including some identified in cancers, stabilized a monomeric state, leading to deficiencies in membrane permeabilization in liposome dye release assays and in cell death induction. These data support the idea that mutations or binding of ligands, such as ATP, within the PsKD favor a monomeric form of hMLKL that exists basally in the cytoplasm prior to the cell receiving a necroptotic stimulus. Using crosslinking MS (XL-MS) to direct molecular modeling, we found the 4HB domain was bound in *cis* by the suppressor PsKD, which thereby restricted its executioner activity. The transition to the hMLKL tetramer was modeled from small-angle X-ray scattering (SAXS) data and molecular dynamics (MD), with intermolecular crosslinks as constraints, yielding a model in which the second brace helix serves an important role in higher-order assembly: an assertion further supported by hydrogen–deuterium exchange MS (HDX-MS).

Previous studies have reported that hMLKL oligomerization, and thus activation, can be promoted by introduction of negatively charged residues to mimic hRIPK3-mediated phosphorylation within the PsKD activation loop, T357 and S358^2,12,18^. However, reconstitution of *MLKL*^*−/−*^ U937 and HT29 cells with hMLKL constructs encoding phosphomimetic (Glu or Asp) mutations of T357 and S358 neither induced constitutive cell death nor reconstituted cell death upon treatment with necroptotic stimuli. These data indicate that, contrary to its mouse counterpart, activation of human MLKL relies on additional steps beyond modification by RIPK3 for necroptotic signaling to ensue. Overall, this work provides insights into how residues within the MLKL PsKD promote MLKL structural change and the self-association essential for necroptosis.

## Results

### hMLKL exists in three conformational states

**Fig. 1:**
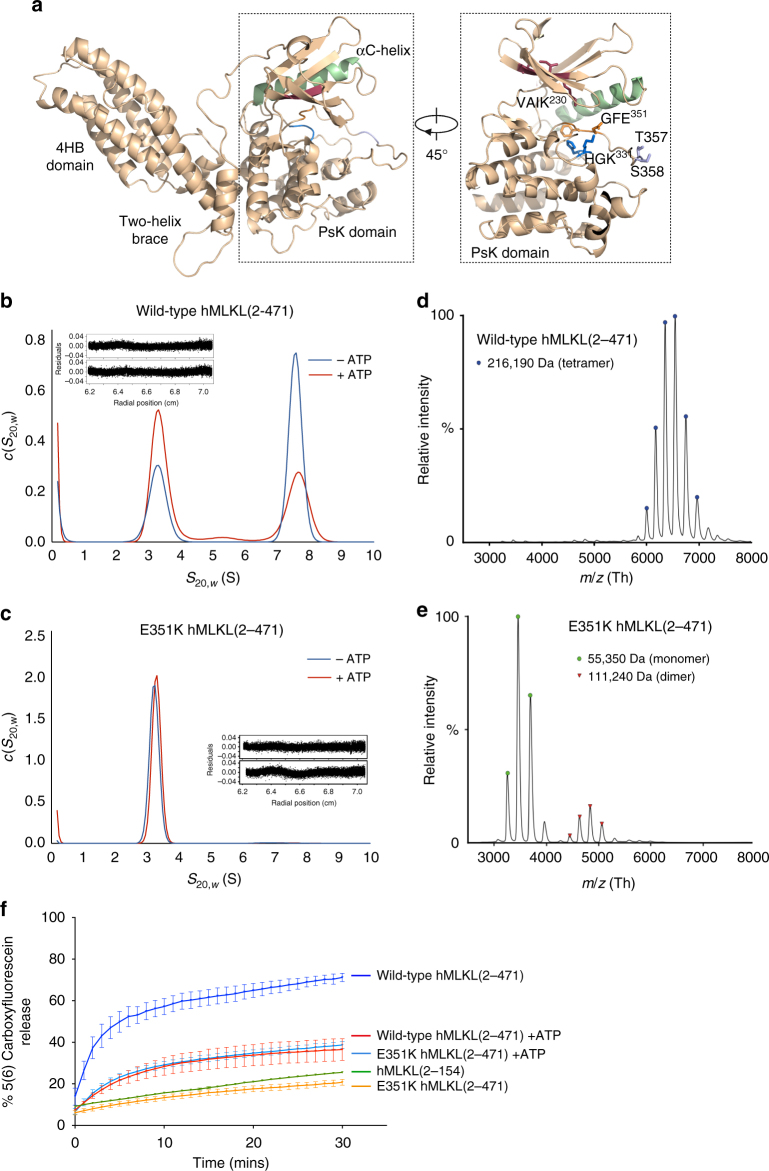
Tetrameric hMLKL(2–471) is destabilized by ATP or PsK domain mutation to a monomeric intermediate. **a** Homology model of hMLKL^21^. The αC helix is highlighted green, the VAIK^230^ maroon, HGK^331^ blue, GFE^351^ orange, and the RIPK3 phosphorylation site, Thr357/Ser358, in purple. **b**, **c** Standardized continuous sedimentation coefficient [*c*(*s*_20,w_)] distribution of wild-type and E351K hMLKL(2–471) in the presence (red) and absence (blue) of 300 μM ATP at 1 mg/mL. Residuals for the best fit of the raw radial absorbance sedimentation velocity data to a continuous sedimentation coefficient [*c*(*s*)] distribution model are shown as an inset for corresponding protein at 1 mg/mL in the absence of ATP (top) and presence of 300 μM ATP (bottom). **d**, **e** Mass spectra of wild-type and E351K hMLKL(2–471) under native conditions. **f** Liposomes containing self-quenching dye 5(6)-carboxyfluorescein were added to 1 μM of wild-type hMLKL (dark blue), wild-type hMLKL + ATP (red), E351K hMLKL (orange), E351K hMLKL + ATP (light blue), and hMLKL(2-154) (green), and dye release was monitored by spectrophotometry at 485 nm over 30 min. Data represent mean ± SEM of three independent assays

In cells, cytosolic MLKL is monomeric prior to phosphorylation within the pseudoactive site by RIPK3^7,11^. This led us to hypothesize that rearrangement within the PsKD, induced by phosphorylation of the activation loop, drives oligomer formation in vivo. Recombinant full-length hMLKL has been reported to exist in an oligomer:monomer equilibrium^12^, suggesting a dynamic interconversion in solution. E351 is part of the GFE motif present in MLKL orthologs, which is the equivalent of the Mg^2+^-binding DFG motif in active kinases. E351 resides adjacent to activation loop RIPK3 substrate residues, T357 and S358, proximal to other features common to kinases: the VAIK^230^ motif and catalytic loop HRD motif counterpart (HG^330^K^331^) (Fig. 1a). We previously observed that introduction of an E351K mutation, which was first identified in a lung carcinoma^19^, into the recombinant PsKD led to enhanced ATP binding^14^. To determine whether enhanced ATP binding within the PsKD might perturb the oligomer:monomer equilibrium, we compared full-length E351K and wild-type hMLKL. While wild-type hMLKL eluted as two distinct peaks from size exclusion chromatography (SEC), as expected^12^, E351K eluted as a single species at a retention time equivalent to the 55 kDa wild-type hMLKL monomer (Supplementary Fig. 1a, b). We analyzed the self-association of full-length wild-type and E351K hMLKL at 1, 0.5, and 0.25 mg/mL using sedimentation velocity (SV) analytical ultracentrifugation (AUC). At each concentration, two species were observed for wild-type hMLKL with sedimentation coefficients of 3.5 S and 8.2 S, while only one 3.5 S species was observed for E351K (Supplementary Fig. 1c, d). While the addition of 300 μM ATP did not alter the positions of either protein at 1 mg/mL, ATP skewed the equilibrium of wild-type hMLKL toward the smaller 3.5 S species (Fig. 1b). Under these conditions, E351K remained monomeric (Fig. 1c).

To accurately determine the molecular weight of the higher-order species of MLKL, we performed native MS analysis on the wild-type hMLKL oligomer SEC fraction. The mass spectrum revealed a charge envelope consistent with a tetrameric protein complex (216,190 Da) (Fig. 1d). In contrast, the most abundant species in the E351K mass spectrum corresponds to a monomer (55,350 Da) in addition to a very small population of dimer (111,240 Da) (Fig. 1e). These results confirm that hMLKL forms a tetrameric complex, consistent with the larger species observed by SV-AUC, which is disrupted by the introduction of the E351K mutation in the PsKD.

Bilayer permeabilization driven by wild-type and E351K hMLKL was measured using a liposome dye release assay^7^. E351K and the N-terminal domain (hNTD; comprising residues 2–154) induced modest dye release (~15% of maximum) (Fig. 1f), while wild-type hMLKL caused rapid 5(6)-carboxyfluorescein release to ~80% of maximum dye release. Addition of ATP to the system enhanced E351K and lowered wild-type hMLKL activity to converge at ~40% maximum dye release (Fig. 1f). Taken together with the AUC and native MS results, the activity of these proteins in the liposome dye release assays indicates that recombinant hMLKL forms a tetrameric structure in solution that is destabilized by conformational changes induced by ATP binding or mutations in the vicinity of the PsKD activation loop. Moreover, the liposome permeabilization activity of E351K hMLKL suggests that MLKL can transition between three distinct conformational states: a basal monomer, a nucleotide-loaded transition-state monomer with intermediate liposome permeabilization activity, and the necroptotic tetramer.

### The 4HB domain packs against the PsKD αC helix in monomeric hMLKL

**Fig. 2:**
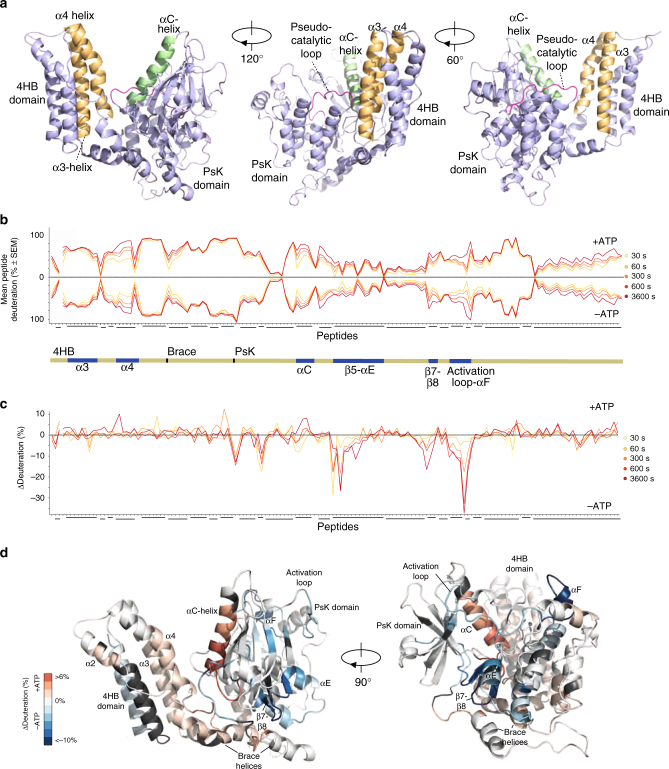
4HB packs against the αC helix in the hMLKL PsKD and is displaced upon ATP binding. **a** E351K hMLKL(2–471) structure modeled using molecular dynamics based on distance restraints defined by DMTMM crosslinking experiments (Supplementary Fig. 2a, Supplementary Data 1). The αC helix is highlighted in green, while α3 and α4 of the 4HB domain (yellow) pack against the αC helix. The RIPK3 substrates, Thr357 and Ser358, are shown as sticks. **b** Mirror plot demonstrating deuteration (percentage of theoretical maximum) of E351K hMLKL peptides±ATP over the indicated time points. Error bars represent SEM of triplicate experiments. Each tick on the *x* axis represents an individual peptide. Overlapping or adjacent peptides were identified for all regions of the protein except for a small section of the N-terminal 4HB. A line break in this region has been introduced to illustrate the gap in sequence coverage (see Supplementary Fig. 3a for peptide sequence coverage). Overlapping regions of peptide coverage are illustrated using tiled lines below the *x* axis. All SEM values did not exceed ±5.96 from *n* = 3 independent time course experiments; error bars omitted for clarity. **c** Differential deuterium uptake of E351K hMLKL peptides ±ATP over the indicated time points (calculated + ATP deuteration minus -ATP deuteration). As in **b**, each tick represents an individual peptide with a line break introduced to illustrate a gap in sequence coverage. Peptides corresponding to overlapping sequences are shown as tiles below the plot. The corresponding regions of hMLKL the peptides map to are shown schematically between **c** and **d**, with sequence elements that undergo differential exchange±ATP highlighted in blue. **d** Differential hydrogen–deuterium exchange (E351K hMLKL±ATP, 60 min time point) for each analyzed peptide was averaged across overlapping sequences per amino acid residue (excluding the first 2 residues of each peptide due to high rates of back exchange) and mapped onto the E351K hMLKL model (see Supplementary Fig. 3b). Dark gray coloring represents regions of the protein excluded from analysis owing to insufficient sequence coverage

We hypothesized E351K hMLKL reflects the basal monomeric conformation of cytosolic hMLKL prior to necroptotic stimuli and sought to model the structure using a chemical XL-MS approach. Using 4-(4,6-dimethoxy-1,3,5-triazin-2-yl)-4-methylmorpholinium chloride (DMTMM) coupling, “zero-length” crosslinks between proximal aspartic/glutamic acid and lysine side chains^20^ were identified by MS (Supplementary Data 1, Supplementary Fig. 2a). From an initial open model of hMLKL^21^ (Fig. 1a), generated using the hMLKL N-terminal domain nuclear magnetic resonance structure^13^ and PsKD crystal structure^14^ by homology with the crystal structure of full-length mouse MLKL^1^, the lowest-energy large-amplitude normal mode that brought the 4HB and PsKDs into contact with one another was traced. This unbiased domain reorientation yielded a structure in good agreement with the chemical crosslinking results. High-scoring links corresponded to Cα–Cα separations less than the upper-bound limit of 30 Å (Supplementary Data 1). Thus the resulting model reveals a compact structure in which the 4HB domain is packed against the αC helix and pseudocatalytic loop of the PsKD (Fig. 2a).

Addition of ATP to E351K hMLKL resulted in a transition to a conformer with increased ability to disrupt liposomes (Fig. 1f). To analyze changes in secondary structure (via hydrogen bonding) and dynamics of this transition from a basal to a pro-necroptotic state, we used HDX-MS in the presence or absence of ATP. ATP binding to E351K hMLKL resulted in a reduction of deuterium exchange in the regions surrounding the ATP-binding pocket in the PsKD (Fig. 2b–d; Supplementary Fig. 3) and increased in deuterium exchange in the PsKD αC helix (Arg-244 to Leu-263) and in the α3 and α4 helices from the 4HB domain that abut the αC helix in our model (Fig. 2b–d). This suggests that, upon ATP binding, the 4HB and αC helix become disengaged leading to an increase in secondary structure dynamics and a reduction in stability of the α-helices in each domain as the protein transitions to a pro-necroptotic conformation.

### hMLKL tetramer assembles into a daisy chain configuration

**Fig. 3:**
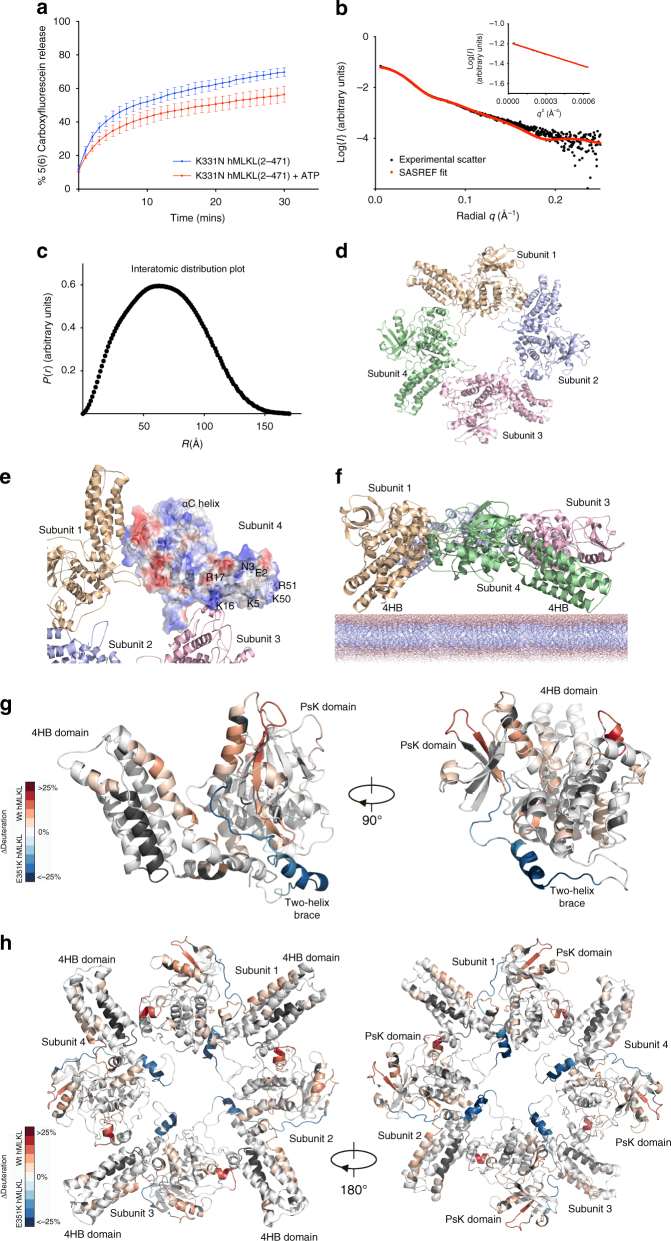
hMLKL(2–471) tetramerizes in a daisy-chain arrangement. **a** Liposome dye release assay of K331N hMLKL(2–471) at 1 μM in the absence (blue) and presence (red) of 500 μM ATP. Dye release was monitored by spectrophotometry at 485 nm over 30 min. Data represent mean ± SEM of three independent assays. **b** Small angle scattering profile of averaged and background subtracted data from the apex of inline size exclusion peak (black circles). Agreement between the experimental and theoretical scatter (red circles) calculated from a rigid body tetramer model in SASREF is reflected in the *χ* value of 0.54. Inset: Guinier plot of experimental SAXS data for *q*.Rg < 1.3, illustrating that aggregated species do not contribute substantively to the scatter. **c** Interatomic distance distribution plot, *P*(*r*), profile calculated from scattering data with GNOM. Maximum particle dimension, *D*_max_, was estimated as 170 Å. **d** Arrangement of subunits within the hMLKL tetramer. **e** Electrostatic surface of one subunit in the tetramer. Charge distribution is consistent with available presentation of residues with positive charge in the 4HB domain to engage lipid (lipid-binding residues defined in ref. ^17^ are labeled). **f** Model of tetramer binding to lipid bilayer, where the planar presentation of the four N-terminal domains suggests simultaneous engagement. **g**, **h** Differential hydrogen–deuterium exchange (wild-type vs E351K hMLKL, 5 min time point) for each analyzed peptide was averaged across overlapping sequences for each amino acid residue (excluding the first three residues of each peptide due to high rates of back exchange) and mapped onto the crosslink-refined E351K hMLKL monomer model (**g**) or the hMLKL tetramer model shown with 4HB down (left) or PsK down (right; **h**). Dark gray coloring represents regions of the protein excluded from analysis owing to insufficient sequence coverage

In previous mutational studies of the human MLKL PsKD, we observed that introduction of a K331N substitution in the pseudocatalytic loop decreased ATP affinity^14^. Here, in the context of full-length hMLKL, K331N substitution markedly favored tetramer formation over monomer by SV-AUC sedimentation experiments (Supplementary Fig. 4a), thus identifying K331N hMLKL(2–471) as an ideal candidate for biophysical characterization of the hMLKL tetramer. Recombinant K331N permeabilized liposomes equivalently to wild-type hMLKL in the absence of ATP, with only modest perturbation by the addition of ATP (Fig. 3a), indicating that this mutation promotes a hMLKL conformer that is predisposed to forming higher-order assemblies. To define the in-solution structure of the hMLKL tetramer, we performed SAXS analysis on recombinant K331N hMLKL eluted by inline SEC into the path of the X-ray beam (statistics shown in Supplementary Table 1). Scatter profiles corresponding to the apex of the tetramer SEC peak were averaged and background subtracted, with Guinier analysis indicating that the data were monodisperse (Fig. 3b) with a maximum particle dimension (*D*_max_) of 170 Å (Fig. 3c). We fitted a tetrameric model of hMLKL to these scattering data by rigid-body modeling of the reported hMLKL model^21^ using maximum 35 Å distance restraints between K157–K157, K173–K173 and K305–K305 pairs in neighboring protomers (Fig. 3d). These contacts were experimentally derived from crosslinks identified in hMLKL tetramers by MS following treatment with the primary amine crosslinkers, disuccinimidyl suberate (DSS) and Bis(sulfosuccinmidyl) suberate (BS^3^) (Supplementary Fig. 2b, c; Supplementary Data 1). These restraints are few, because only the like-to-like crosslinks mediated by DSS and BS^3^ could be unambiguously assigned as intersubunit linkages within the hMLKL tetramer. Molecular dynamics refinement and simulated annealing minimization were used to further refine the tetramer model. Theoretical scatter calculated for our model was consistent with the experimental data (*χ* = 0.542 using CRYSOL; Fig. 3b). The tetramer model (Fig. 3d) revealed an arrangement of protomers in which residues previously reported to be involved in lipid engagement^17^ were surface exposed (Fig. 3e, f) and all 4HB domains presented in a plane that would allow simultaneous lipid engagement (Fig. 3e, f) The daisy chain-like tetramer arrangement of hMLKL formed through heterologous interfaces, rather than a dimer of dimers with isologous interfaces, is consistent with the AUC and native MS analyses, which suggest that the protein exists in monomer and tetramer states but not dimers and trimers.

To understand the dynamic changes that occur to MLKL upon tetramer formation, we performed HDX-MS on the E351K (monomer) and wild-type (tetramer) hMLKL proteins (Fig. 3g, h; Supplementary Fig. 2d–f). We observed markedly reduced deuterium exchange in wild-type relative to E351K hMLKL in the brace region (residues 151 and 194) (Fig. 3h) and increased deuterium exchange in the PsKD αC helix and in the 4HB domain α4 helix within the tetramer (Fig. 3g), consistent with 4HB domain reorientation away from the PsKD within the tetramer.

### PsKD switch mutations compromise liposome permeabilization

**Fig. 4:**
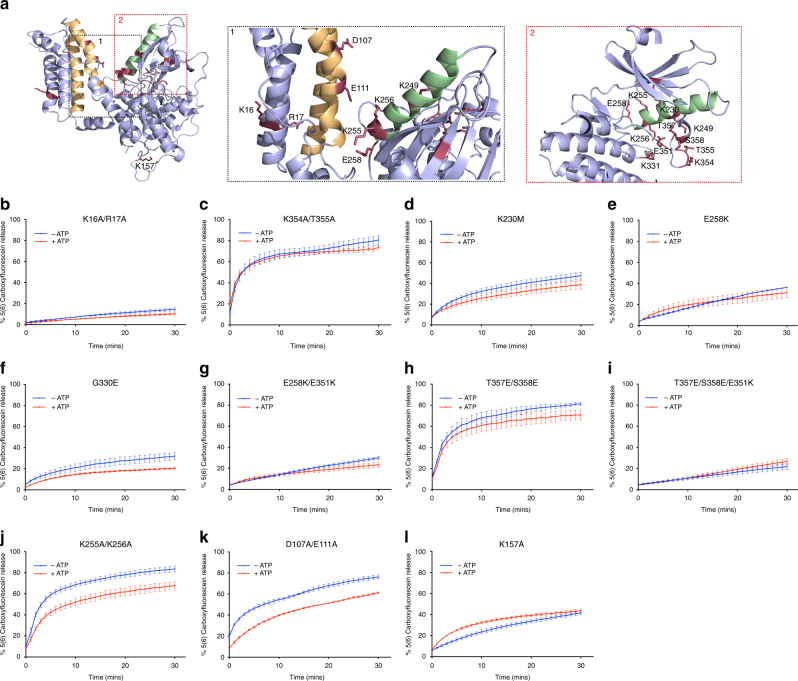
hMLKL(2–471) liposome permeabilization activity is affected by PsK domain mutations that disrupt the molecular switch. **a** Residues mutated in the present study are shown as sticks on the E351K hMLKL model generated in the course of this study (Fig. 2a), with zoomed views of the 4HB-PsK interface (panel 1) and the pseudoactive site in the PsK domain (panel 2) shown on the right. **b**–**l** 5(6)-Carboxyfluorescein egress from liposomes induced by 1 μM full-length hMLKL in the presence (red) and absence (blue) of 500 μM ATP was monitored by absorbance at 485 nm for K16A/R17A (**b**), K354A/T355A (**c**), K230M (**d**), E258K (**e**), G330E (**f**), E258K/E351K (**g**), T357E/S358E (**h**), T357E/S358E/E351K (**i**), K255A/K256A (**j**), D107A/E111A (**k**), and K157A (**l**) mutants. Data represent mean ± SEM of three independent assays

To understand how changes in the PsKD of MLKL control the transition between monomer and tetramer, we introduced a series of mutations into full-length hMLKL guided by XL-MS, HDX-MS, and residues mutated in human cancer specimens (Fig. 4a, Table 1). Mutations in hMLKL PsKD annotated in human tumors are of interest because defective necroptosis may underlie persistence of tumors. To benchmark activities in liposome permeabilization assays, we compared recombinant wild-type or mutant full-length hMLKL to a previously reported control mutant, K16A/R17A hMLKL, which exhibits compromised liposome permeabilization^17^ (Fig. 4b).

**Table 1:** Summary of wild-type and mutant hMLKL properties

hMLKL mutation	Location	Liposome permeabilization –ATP/+ATP	ATP-binding *K*_d_ (±SEM; μM)	Necroptotic killing function	Tumor association
Nil (wild type)		+++/++	36 ± 4	+	–
K230M	PsK β3 strand	++/++	–	Delayed	K230Q in colon carcinoma
K255A/K256A	PsK αC helix	+++/++	ND	+	–
E258K	PsK αC helix	+/+	24 ± 2	Delayed	E258K in colon and endometrial carcinomas^19^
G330E	PsK catalytic loop	+/+	28 ± 6	Delayed	G330E melanoma; G330R endometrial carcinoma^19^
K331N	PsK catalytic loop	+++/+++	–	+	–
E351K	PsK activation loop	+/++	26 ± 6	Delayed	E351K lung carcinoma^19^; E351Q prostate adenocarcinoma
K354A/T355A	PsK activation loop	+++/+++	−	ND	−
T357E/S358E	PsK activation loop	+++/+++	56 ± 8	−	−
K16A/R17A	4HB α1 helix	+/+	ND	Delayed	R17W in endometrial cancer^19^
D107A/E111A	4HB α4 helix	+++/++	23 ± 2	−	−
K157A	Brace helices	++/++	164 ± 31	Delayed	−

ND not determined; −, not detected

The activation loop of the hMLKL PsKD contains E351 of the GFE (“DFG”) motif, K354/T355, and the RIPK3 substrates, T357 and S358 (Fig. 4a). In our model, E351 is surrounded by other residues of interest: K230 of the VAIK motif, which positions ATP for catalysis in active protein kinases and was previously implicated in hMLKL PsKD ATP binding^14^; G330 of the HGK motif (equivalent to the catalytic “HRD” motif in active kinases); and the αC helix residue, E258. Mutation of each of these residues led to deficits in liposome permeabilization (Fig. 4), which could not be overcome by composite mutations, such as E258K/E351K. E258 formed DMTMM crosslinks with K351 within the E351K hMLKL monomeric mutant (Supplementary Fig. 2a, Supplementary Data 1), indicative of a salt bridge present in these mutants that locks the protein in a suppressed conformation.

Phosphorylation of the activation loop residues, T357 and S358, by RIPK3 or replacement with phosphomimetic Asp or Glu residues was reported to promote oligomer formation of hMLKL^2,12^. We introduced phosphomimetic Glu residues into both wild-type and E351K hMLKL. T357E/S358E hMLKL behaved similarly to wild-type hMLKL except that its ability to permeabilize liposomes was not diminished in the presence of ATP. Alanine substitution of the adjacent K354/T355 led to an hMLKL with similar behavior in liposome assays (Fig. 4c), with similar lack of attenuation upon ATP. The effects of activation loop substitution are consistent with the idea that phosphomimetic mutations, and perhaps phosphorylation itself, stabilize MLKL tetramers and ATP does not induce dissociation of this stabilized form (Fig. 4h). Somewhat surprisingly, T357E/S358E/E351K hMLKL was unable to disrupt liposomes and was unresponsive to ATP, indicating a block in the molecular switch-driven transition between the three conformational states of hMLKL (Fig. 4i).

In both the wild-type and E351K hMLKL monomers, a pair of lysines adjacent to the aforementioned E258 in the αC helix, K255 and K256 (Fig. 4a), were observed to form zero-length (DMTMM) crosslinks with E111 in the 4HB domain (Supplementary Data 1). These crosslinks were not detected in the wild-type hMLKL tetramer, raising the possibility that K255/K256 contribute to maintaining the monomeric conformation. The K255A/K256A and D107A/E111A hMLKL mutants exhibited liposome permeabilization comparable to wild-type hMLKL (Fig. 4j, k). Liposome permeabilization by these mutants was only modestly impacted by ATP and, to comparable extents, consistent with reciprocal interaction of these sites within the 4HB domain-restrained monomeric conformation (Fig. 4j, k). Deficits in liposome permeabilization observed among PsKD mutants were not attributable to compromised lipid binding, because E258K and G330E hMLKL bound membrane lipid arrays with a preference for phosphatidylserine, phosphatidylinositol-4-phosphate, and cardiolipin, like wild-type and D107A/E111A hMLKL (Supplementary Fig. 5), and consistent with similar reports for wild-type hMLKL^10,12^.

Our recent studies have implicated the brace helices in communicating activating signals from the PsKD to the executioner 4HB domain and as an important contributor to MLKL oligomerization^22^. Mutation of brace residue, K157, which formed DSS/BS^3^ crosslinks to K157 of another protomer within the wild-type hMLKL tetramer (Supplementary Fig. 2a, b), induced deficits in liposome permeabilization (~40% dye egress vs 80% for wild-type hMLKL; Fig. 4l).

### PsKD mutations restrict molecular switch toggling by ATP

The observation that ATP binding destabilized full-length wild-type hMLKL tetramer formation and attenuated liposome permeabilization activity led us to examine whether ATP binding might restrict MLKL activation. Using a thermal stability shift assay^16,23^, we measured the affinity of full-length wild-type and mutant hMLKL for ATP (Table 1; Supplementary Fig. 4). While wild-type hMLKL bound ATP with a *K*_d_ of 36 μM, curiously, the liposome permeabilizing activities of T357E/S358E, E258K, and G330E hMLKL were unaffected by ATP (Fig. 4) despite comparable affinities, suggesting that these mutations perturb (or override) any conformational change that may impact MLKL activation upon ATP binding. In contrast, E351K hMLKL bound ATP with a *K*_d_ of 26 μM, and ATP binding induced elevated liposome permeabilization activity (Fig. 1f), consistent with a molecular switch that can be toggled by ATP in E351K hMLKL to relieve the repression of hMLKL activation. Unsurprisingly, K230M, K331N, K354A/T355A, and T357E/S358E/E351K hMLKL did not markedly respond to ATP in the liposome dye release assays, consistent with their ATP-binding deficits (Supplementary Fig. 4). Overall, residues within the PsKD play key roles in governing hMLKL activation, and their mutation can severely impact (or override) the sensitivity of hMLKL to ATP-binding-induced conformational changes. We expected mutations outside the PsKD to have little impact on ATP binding. Although true of D107A/E111A hMLKL, surprisingly, K157A hMLKL bound ATP with the relatively weak *K*_d_ ATP of 164 μM, which led to minimal impact of ATP on K157A hMLKL liposome permeabilization.

### Monomer promoting PsKD mutations delay necroptosis

**Fig. 5:**
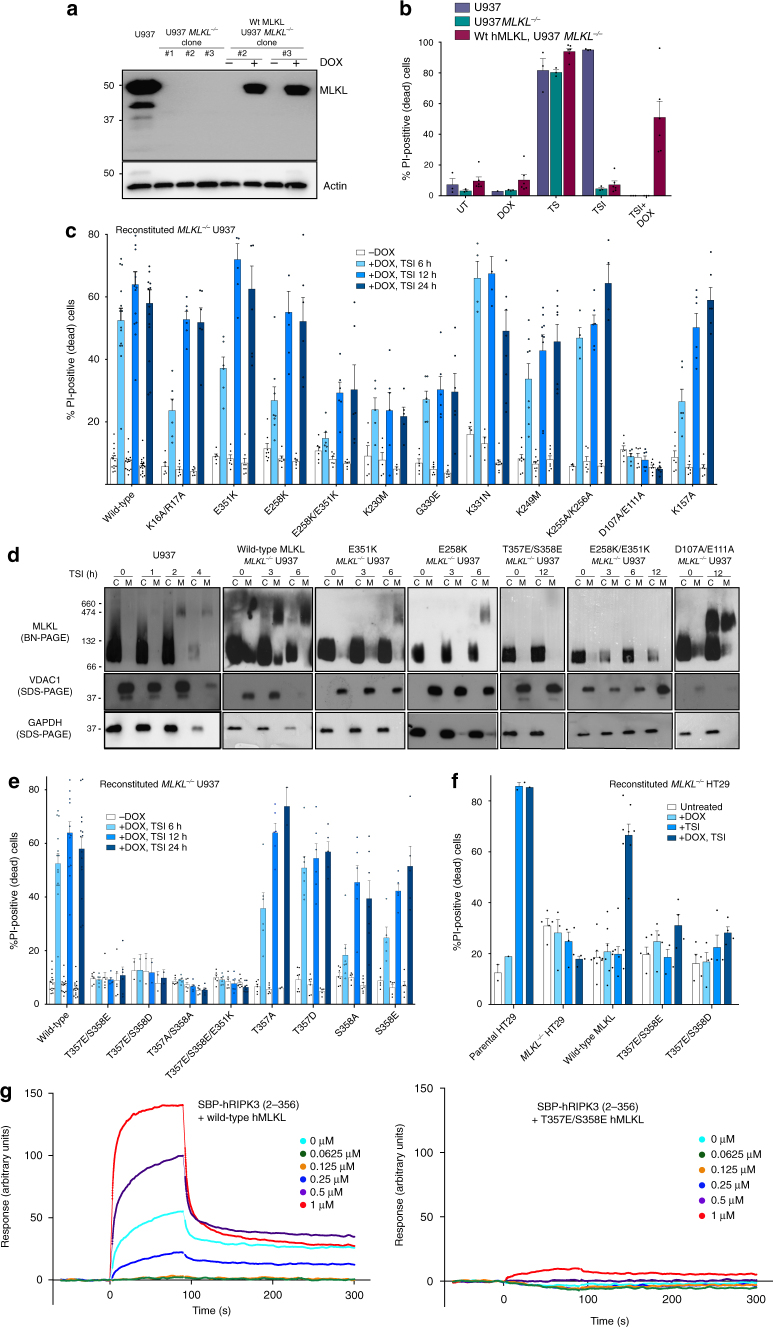
Mutations in the PsK domain compromise hMLKL-driven necroptosis in cells. **a** MLKL expression in parental U937, edited *MLKL*^*−/−*^ U937, and *MLKL*^*−/−*^ U937 cells reconstituted with a doxycycline-inducible wild-type hMLKL construct were assessed by western blot. Loading control: anti-actin reprobe. **b** Sensitivity of parental U937, edited *MLKL*^*−/−*^ U937, and *MLKL*^*−/−*^ U937 reconstituted with doxycycline-inducible wild-type hMLKL to apoptotic (TS) and necroptotic (TSI) stimuli was assessed by PI uptake and flow cytometry. Data represent mean ± SEM of three independent assays. **c** Sensitivity of *MLKL*^*−/−*^ U937 cells reconstituted with doxycycline-inducible wild-type or mutant hMLKL to necroptotic death assessed at 6, 12, and 24 h post-TSI treatment by PI uptake and flow cytometry. Individual data points are plotted as circles. **d** Parental U937 and *MLKL*^*−/−*^ U937 reconstituted with wild-type hMLKL or the E351K, E258K, T357E/S358E, E258K/E351K, or D107A/E111A mutants were assessed for oligomer formation by Blue Native PAGE post-TSI treatment at the indicated time points. Separation into cytoplasmic (C) and membrane (M) fraction was validated by SDS-PAGE western blots for VDAC1 (membrane) and GAPDH (cytoplasmic). All blots are representative of ≥2 independent experiments. **e** Sensitivity of *MLKL*^*−/−*^ U937 cells reconstituted with doxycycline-inducible wild-type or mutant T357E/S358E, T357E/S358D, T357A/S358A, T357E/S358E/E351K, T357A, T357D, S358A, and S358E hMLKL to necroptotic death assessed at 6, 12, and 24 h post-TSI treatment by PI uptake and flow cytometry. Data in **c** and **e** represent mean±SEM of two independent assays of duplicate cell lines, except for T357E/S358D, which represents mean ± SD of two independent assays on a single line. Individual data points are plotted as circles. **f** Sensitivity of parental HT29, *MLKL*^*−/−*^ HT29, and *MLKL*^*−/−*^ HT29 cells reconstituted with doxycycline-inducible wild-type or mutant T357E/S358E, T357E/S358D hMLKL to necroptotic death at 48 h post-TSI treatment. Data represent mean±SEM of two independent assays on 4 (wild-type) or 2 (mutant) cell lines. Data for parental HT29 cells from a single experiment are shown for comparison. Individual data points are plotted as circles. **g** Streptavidin-binding peptide (SBP)-tagged hRIPK3 kinase domain immobilized on a streptavidin chip showed robust binding to wild-type, but not T357E/S358E, hMLKL (analyte applied over 0–1 μM) by surface plasmon resonance. Data are representative of four independent experiments

Having identified residues in the hMLKL PsKD as important determinants of activation in in vitro assays, we sought to establish their contribution to cellular necroptosis signaling. To this end, we generated *MLKL*^*−/−*^ U937 cells by CRISPR editing using a non-integrating lentivirus and reconstituted the necroptosis pathway by expressing wild-type or mutant hMLKL for 16 h via a doxycycline-inducible lentiviral vector before sensitivity to death stimuli was assessed. *MLKL* knockout was verified by western blot (Fig. 5a) and next-generation sequencing, and manifested in negligible cell death (Fig. 5b), as quantified by propidium iodide (PI) uptake using flow cytometry, following stimulation with the necroptotic stimulus, TSI (comprising tumor necrosis factor (TNF) (T), the Smac mimetic Compound A (S), and the pan-caspase inhibitor, emricasan/IDN-6556 (I); as reported previously^22,24^). Induced expression of wild-type hMLKL in *MLKL*^*−/−*^ U937 cells restored sensitivity to the necroptotic stimulus, TSI, resulting in ~55% cell death 24 h posttreatment (Fig. 5b, c). We further validated this cellular system as a platform for examining the impact of mutating MLKL on necroptotic signaling by introducing the mutant, K16A/R17A hMLKL. Consistent with an earlier study^17^, K16A/R17A hMLKL exhibited retarded cell death relative to wild-type hMLKL, with reduced cell death observed at 6 h but comparable death to wild-type hMLKL at 12 and 24 h time points (Fig. 5c). Importantly, wild-type and all mutant hMLKL constructs examined herein were expressed at equivalent levels, as assessed by western blot analysis (Supplementary Fig. 6), indicating that any deficits in necroptotic signaling are not a consequence of reduced protein expression. All cell lines showed comparable sensitivity to the apoptotic stimulus, TS, in the presence and absence of doxycyline-induced protein expression. As expected, doxycycline was essential for responsiveness to the necroptotic stimulus, TSI, in reconstituted *MLKL*^*−/−*^ U937 lines (Supplementary Fig. 7).

Pseudoactive site integrity was important to hMLKL activity in liposome assays (Fig. 4), and mutations to E351, E258, G330, and K230 have been observed in various tumors (Table 1), raising the possibility that their mutation may compromise necroptosis. Consistent with this idea, relative to wild-type hMLKL, expression of E351K or E258K hMLKL in *MLKL*^*−/−*^ U937 cells (Fig. 5c), but not parental U937 cells (Supplementary Fig. 7j, l), led to delayed kinetics of necroptotic death comparable to K16A/R17A hMLKL. This was borne out in Blue Native polyacrylamide gel electrophoresis (BN-PAGE) analyses, where hallmarks of hMLKL activation—translocation of hMLKL from cytoplasm (C) to membrane (M) fraction, the membrane fraction assembling into higher-order species and undergoing phosphorylation on the activation loop residue S358—was delayed for the E258K hMLKL mutant relative to wild type (Fig. 5d; Supplementary Fig. 8). While wild-type hMLKL was phosphorylated, oligomerized, and membrane translocated robustly from 4 h post-stimulation, E258K hMLKL was only modestly oligomerized and membrane translocated, with a correspondingly weak phospho-S358 signal detected (Supplementary Fig. 8). We concluded that, because E258K hMLKL was phosphorylated, albeit weakly over this timeframe, it has retained the capacity to engage RIPK3. Comparatively, E258K/E351K hMLKL was even more defective, with maximal ~50% death observed at 24 h post-TSI. E258K/E351K hMLKL exhibited attenuated membrane translocation, higher-order hMLKL complex assembly and no S358 phosphorylation by BN-PAGE post-TSI up to 10 h post-stimulation, while robust signals were observed for the wild-type hMLKL counterpart at 4 h post-stimulation (Fig. 5c, d; Supplementary Fig. 8). Defective necroptosis was similarly profound in the K230M and G330E mutants where levels of death were only ~50% of those of wild-type hMLKL at 24 h post-stimulation (Fig. 5c), while the neighboring mutant, K331N, exhibited no deficits in necroptotic signaling (Fig. 5c). We observed that the αC helix undergoes enhanced solvent exchange in monomeric E351K hMLKL upon addition of ATP and accordingly examined the role of the proximal αC helix residue, K249 (Fig. 4a), as a probe for conformational changes induced by the molecular switch mechanism. Because of the importance of K230 and E351 to MLKL activation, it was unsurprising that mutation of the proximal αC helix residue, K249, also led to delayed necroptotic signaling (Fig. 5c).

In our MLKL monomer model arising from XL-MS, the αC helix residues, K255 and K256, are adjacent to D107 and E111. We examined the contributions of these contacts to hMLKL activation by reconstituting *MLKL*^*−/−*^ U937 cells with K255A/K256A hMLKL or D107A/E111A hMLKL. While mutation of K255/K256 were dispensable, the integrity of D107/E111 proved essential to hMLKL-mediated cell death. Membrane-associated high molecular weight assemblies are a hallmark of hMLKL activation, but interestingly, despite failing to signal for cell death, D107A/E111A hMLKL assembled into the higher molecular complexes in both cytoplasmic and membrane fractions (Fig. 5d), and curiously S358 was phosphorylated in both fractions (Supplementary Fig. 8). D107 and E111 are the hMLKL counterparts of D106 and E110 previously implicated as essential for mouse MLKL-mediated necroptosis^11^, indicating that these residues play conserved roles in recruiting necroptotic co-effectors required for MLKL translocation to, and/or permeabilization of, membranes. The identities of these additional factors remain of outstanding interest.

In addition to K157-K157 BS^3^/DSS crosslinks between subunits within the hMLKL tetramer, K157 formed zero-length crosslinks with D300 in the C-lobe of the PsKD, suggesting a possible role in both communicating conformational changes from the PsK to the 4HB domain. Consistent with the deficits observed in liposome permeabilization assays, K157A hMLKL exhibited delayed necroptosis kinetics in reconstituted *MLKL*^*−/−*^ U937 cells, with only ~50% of the death observed for wild-type hMLKL at 6 h (Fig. 5c).

### Phosphomimetic PsKD mutants do not induce necroptosis

**Fig. 6:**
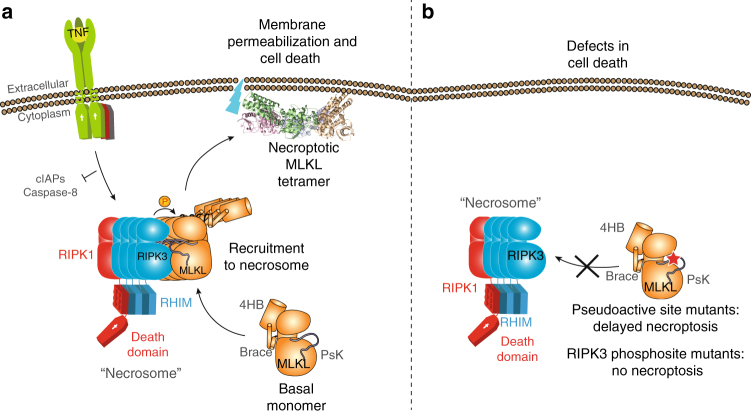
The necrosome recruits hMLKL via RIPK3 before RIPK3-mediated phosphorylation induces hMLKL reorganization and/or disengagement, membrane translocation, and necroptotic death. **a** A revised model for MLKL activation. Human MLKL relies on recruitment to the necrosome via RIPK3, which scaffolds assembly of necroptotic MLKL tetramers and their reorganization and/or disengagement following RIPK3-mediated phosphorylation. **b** In contrast to mouse MLKL, which can be activated by mutations that mimic RIPK3-mediated phosphorylation, hMLKL activity is compromised by pseudoactive site and activation loop mutations

In contrast to mouse MLKL^1,11^, introduction of point mutations in the hMLKL PsKD did not induce stimulus-independent death but rather led to defective necroptotic activity (Fig. 5c, d). To further probe the differences in activation mechanisms between mouse and human MLKL, we introduced phosphomimetic (acidic) or phosphoablating (alanine) residues at T357 and S358 in the hMLKL PsK activation loop to, respectively, emulate or block the key activation step, phosphorylation by RIPK3. Introduction of the phosphomimetic substitution, S345D, into mouse MLKL to emulate RIPK3-mediated phosphorylation leads to robust stimulus-independent death in mouse cells^1,8,25^. By contrast, not only did phosphomimetic T357E/S358E and T357E/S358D or phosphoablating T357A/S358A hMLKL mutants fail to induce constitutive cell death, each could not reconstitute necroptosis signaling in *MLKL*^*−/−*^ U937 (and HT29) cells and no cell death was observed 24 (and 48) hours post-stimulation with TSI (Fig. 5e). This was further reflected in an absence of detectable T357E/S358E hMLKL oligomerization and membrane translocation by BN-PAGE at 12 h post-TSI stimulation (Fig. 5d). Notably, similar results were obtained using *MLKL*^*−/−*^ HT29 cells, where inducibly expressed wild-type, but not T357E/S358E or T357E/S358D, hMLKL constructs could reconstitute the necroptosis signaling pathway (Fig. 5f). Furthermore, recombinant wild-type, but not T357E/S358E, hMLKL robustly bound immobilized Streptavidin-binding peptide (SBP)-tagged hRIPK3 kinase domain in surface plasmon resonance (SPR) experiments (Fig. 5g). These data suggest deficits in necrosome recruitment likely underlie defective necroptotic signaling by T357E/S358E hMLKL (Fig. 6). Defective T357E/S358E hMLKL function is not attributable to the mutation compromising MLKL folding or structure, because the crystal structure of T357E/S358E hMLKL PsKD (residues 190–471) solved to 2.8 Å resolution was comparable to that of wild-type hMLKL PsK (RMSD 0.78 Å) (Supplementary Fig. 4b, c; Supplementary Table 2). Additionally, introduction of T357E/S358E substitutions failed to overcome deficits in necroptotic signaling in E351K hMLKL, with negligible cell death observed 24 h post-stimulation with TSI. Introduction of single phosphomimetic and phosphoablating mutations at T357 or S358, on the other hand, did not preclude reconstitution of necroptotic signaling in *MLKL*^*−/−*^ U937 cells. All single residue mutants were capable of restoring signaling to different extents, with T357D most resembling wild-type hMLKL and mutations to S358 the most deleterious (Fig. 5e). Collectively, these data indicate that, while both phosphorylation sites are required for the complete activity of MLKL, S358 is most crucial.

## Discussion

MLKL is ubiquitously expressed^1,26^ and, as such, mechanisms must exist to prevent MLKL activation and necroptotic cell death in the absence of an upstream trigger. Here we reveal the existence of a basal, monomeric conformer in which the executioner 4HB domain interacts with the PsKD via an interface centered around the αC helix to attenuate its killing activity. Using XL-MS and HDX, coupled with in vitro liposome permeabilization assays and biophysical and cellular studies, we implicated a core of pseudoactive site residues centered around the activation loop and αC helix of the human MLKL PsKD in controlling assembly of hMLKL into the tetramers that mediate necroptotic cell death.

The stoichiometry of the human MLKL oligomer has been a matter of debate in the literature, with trimers, tetramers, hexamers, and octamers variously proposed^6,9–12,27,28^, with few studies using high-resolution approaches. Here we used native MS to define the hMLKL oligomer as a tetramer. Further evidence for hMLKL-forming tetramers was obtained from SAXS data, which in combination with XL-MS and MD, enabled low-resolution modeling of the hMLKL tetramer. These data are consistent with a daisy chain assembly, in which the 4HB domain and brace helices abut the foot of the PsKD C-lobe in the neighboring hMLKL protomer. However, detailed knowledge of the precise intersubunit interactions that govern tetramer assembly await a high-resolution tetramer structure. Nonetheless, within our tetramer model, the four 4HB domains (and component residues implicated in lipid binding) are largely surface exposed and are orientated at the outer torus surface, which would allow simultaneous membrane docking. Although not observed in our studies, we cannot exclude the possibility that higher-order oligomers, such as the octamers reported to arise from tetramers via an interface surrounding the 4HB domain α3-α4 loop residue, C86^27^, may arise from hMLKL tetramers in cells.

Our findings indicate that in solution hMLKL populates either tetramer or monomer forms and suggest an intrinsic difference from mouse MLKL, whose 4HB+brace was found to form homotrimers in our earlier AUC^11^ and SAXS^22^ studies. Interspecies differences in oligomeric status are not surprising considering the evolutionary divergence in oligomeric status of other effectors in other death pathways, including the apoptosome scaffold APAF1^29^. In the case of human MLKL, our observations that ATP binding destabilized the hMLKL tetramer and modulated liposome permeabilization indicate that hMLKL can occupy three distinct states: a monomeric form with attenuated activity, which we propose corresponds to the preactivation form; a tetramer, which corresponds to the form that mediates necroptosis; and an intermediate form, in which the PsKD molecular switch mechanism is locked, preventing communication of changes from the PsKD to the executioner 4HB domain.

Consistent with the importance of the “pseudoactive” site in hMLKL to activation, mutations to K230, E258, G330, and E351 have been identified in tumor genomes. Not only did K230M, E258K, G330E, and E351K mutations compromise hMLKL activity in in vitro liposome permeabilization assays but also necroptotic signaling in reconstituted *MLKL*^*−/−*^ U937 cells. Importantly, it is the network of residues centered on the activation loop and αC helix (Fig. 4a) that underpins hMLKL activation, rather than simply ATP-binding propensity. This parallels the situation in mouse MLKL where mutation of the key ATP-binding residue, K219 (the mouse counterpart of human K230), and a partner residue in the activation loop not involved in ATP binding, Q343, can toggle the molecular switch to induce constitutive activation of mMLKL^1^. In hMLKL, mutation of pseudoactive site residues induce the opposite outcome, giving rise to deficits in hMLKL activation. Together, these studies indicate that ATP-binding propensity is a hallmark of a PsKD that can behave as a conformational switch but that ATP binding itself is not necessarily a physiological modulator of MLKL activation. Rather, mutations that favor maintenance of monomeric forms of human MLKL lead to deficits in cell death as a consequence of delayed engagement and phosphorylation by necrosomal hRIPK3. Because E258K hMLKL can still undergo RIPK3-mediated phosphorylation, this supports the idea that monomeric hMLKL mutants have retained the capacity to engage RIPK3. However, the delayed kinetics of oligomerization and RIPK3-mediated phosphorylation of these mutants are consistent with the notion that they are locked in a pre-necroptotic monomeric conformation in the cytosol, which slows their recruitment to RIPK3 and subsequent activation in cells.

In contrast to our earlier studies of mouse MLKL^1,11^, none of the mutations we introduced into the human MLKL PsKD in the present work led to stimulus-independent cell death. Fundamental differences between the mouse and human necroptosis pathways are not altogether surprising considering 71.5%, 60.5%, and 61% amino acid identity between mouse and human RIPK1, RIPK3, and MLKL, respectively, and the inability of human MLKL to reconstitute the necroptosis pathway in mouse *Mlkl*^*−/−*^ fibroblasts^7^. However, the inability of hMLKL PsKD mutants to induce cell death in our study was surprising in light of previous reports^2,12,18,30–32^. The basis for these differences are unclear, although it is possible that the choice of cell line, expression vector, and mode, and whether endogenous MLKL is present, are contributing factors. Moreover, not only did the T357E/S358E or T357E/S358D hMLKL double mutants fail to induce constitutive cell death when expressed in *MLKL*^*−/−*^ U937 and HT29 cells in our hands but it also completely abrogated sensitivity to necroptotic stimuli. Our earlier studies hinted at this possibility: expression of T357E/S358E hMLKL in parental U937s did not induce constitutive necroptosis but instead led to a dominant-negative effect when cells were treated with necroptotic stimuli^7^, presumably owing to hetero-oligomerization with endogenous hMLKL poisoning assembly of active oligomers. While reconstitution of *MLKL*^*−/−*^ U937 and HT29 cells with T358E/S358E hMLKL led to abrogation of necroptotic killing, individual substitution of S358 with Glu or Ala and T357 with Ala led to defective, but not abrogated, sensitivity to necroptotic stimuli in U937 cells. In contrast to mouse MLKL, where a transient “hit-and-run” interaction with RIPK3 to phosphorylate MLKL is sufficient for its activation and can be emulated by the constitutive killer S345D mutation^1,4,11^, our findings suggest a model in which RIPK3 recruits hMLKL to the necrosome via a stable interaction. This assembly precedes RIPK3-mediated phosphorylation to facilitate hMLKL tetramer assembly, reorganization, or disengagement from the necrosome, to allow translocation to the plasma membrane where permeabilization ensues (Fig. 6a). Defective necroptosis arose upon mutation of the hMLKL PsKD pseudoactive site or RIPK3 substrate sites (Fig. 6b), which could be attributed to deficits in RIPK3-mediated recruitment of hMLKL to the necrosome. In this study, we have unveiled crucial, here-to-fore unrealized, mechanistic differences in activation between mouse and human MLKL. An appreciation of species-dependent differences underlying the PsKD molecular switch mechanism is essential to understanding how MLKL drives necroptosis and will prove invaluable to structure-guided design of modulatory small molecules.

## Methods

### Protein expression and purification

Full-length human MLKL and mutants were expressed in Sf21 insect cells as previously described^7^. Briefly, DNA encoding wild-type (synthesized by DNA2.0)  or mutant hMLKL (residues 2–471) were cloned into a pFastBac-derived vector as in-frame fusions with a TEV protease-cleavable N-terminal GST tag. Bacmids generated using the Bac-to-Bac system (Invitrogen) following transformation of DH10MultiBac *Escherichia coli* cells were used to transfect Sf21 insect cells to generate P1 baculovirus according to the Bac-to-Bac manual (Invitrogen). Following a second viral amplification step, P2 virus (10% v/v) was used to infect 0.5 L Sf21 cultures proteins in 2.8 L Fernbach flasks shaking at 90 rpm, 27 °C for 48 h. Human MLKL N-terminal domain (2–154) was expressed via a pGEX-derived vector encoding an in-frame fusion with an N-terminal, TEV protease-cleavable GST tag in *E. coli* BL21 Codon Plus, as previously described^7^. Cell pellets were resuspended in lysis buffer (20 mM Tris, pH 7.5, 200 mM NaCl, 10% glycerol, 0.5 mM TCEP) and cells lysed via sonication. Cell debris was separated from soluble protein via centrifugation at 45,000 × *g* and lysate filtered with a 0.45 μm filter prior to incubation with pre-equilibrated glutathione agarose (UBPBio) at 4 °C for 1 h with gentle agitation. Beads were then collected via centrifugation and washed exhaustively with lysis buffer and mixed with 200 μg of TEV for 2 h at 20 °C or overnight at 4 °C. The supernatant was filtered through a 0.45-μm filter, concentrated via centrifugal ultrafiltration (30 kDa molecular weight cutoff; Millipore) and loaded onto a Superdex S200 gel filtration column (GE Healthcare) equilibrated in gel filtration buffer (20 mM HEPES pH 7.5, 200 mM NaCl, 5% glycerol). For AUC experiments, glycerol was omitted from the buffer. Proteins eluted from SEC column were assessed for purity by reducing sodium dodecyl sulfate (SDS)-PAGE with SimplyBlue SafeStain visualization (ThermoFisher). Except where indicated, when the protein eluted as oligomer and monomer peaks, the two peaks were pooled. Proteins were concentrated to approximately 5 mg/mL and snap frozen for storage at −80 °C. N-terminally SBP-tagged hRIPK3 (2–356; DNA synthesized by ATUM) was expressed in Sf21 cells via a bacmid generated from pFastBac Htb (Invitrogen), which encodes a TEV protease-cleavable N-terminal His_6_ fusion, using methods analogous to those described for mouse RIPK3^33^.

### Liposome dye release assays

Lipids were purchased from Avanti Polar Lipids (Alabaster, AL, USA) and resuspended in chloroform at 20 mg/mL to emulate a plasma membrane composition (20% POPE, 40% POPC, 10% phosphoinositol, 20% DOPS, 10% POPG), as previously reported^7^. Dried lipids were resuspended in 1 mL 50 mM 5(6)-carboxyfluorescein solutions, vortexed, and sonicated and then liposomes extruded through a 100 nm pore membrane. Prior to use, liposomes were purified from excess 5(6)-carboxyfluorescein using PD-10 column (GE Healthcare) equilibrated with 10 mM HEPES pH 7.5, 135 mM KCl. Samples in the presence of ATP were preincubated with 500 μM ATP on ice for 30 min. For dye release assays, 8 μM of liposomes were mixed with 1 μM protein in Falcon 96-well plates and immediately measured every 60 s (excitation 485 nm, emission 535 nm; HiDex Plate reader) at 20 °C for 30 min. 100% dye release was determined by the incubation of liposomes with 1% Triton X-100 and data were represented as a percentage of maximum release. All assays were performed in triplicate; data are presented as a mean ± SD of three independent assays.

### Native mass spectrometry

Recombinant protein was buffer exchanged into 250 mM ammonium acetate using a Micro Bio-Spin 6 column (Bio-Rad, USA). MS spectra were acquired on a Bruker Maxis II ETD using an off-line nano electrospray ionization source (Bruker, Bremen, Germany). Samples were introduced into the ion source via a nanoflow capillary emitter tip prepared in-house with spray current introduced via a titanium wire in the rear of the capillary. The optimized instrument parameters used were as follows: Spectra rate 0.5 Hz, Capillary voltage 1000 V, Dry Temp 60 °C, Funnel 1 RF 400Vpp, Multipole RF 800Vpp, isCID 0 eV, Quadrupole (Ion Energy 1 eV, Low Mass 1000 m/z), Collision Cell (Energy 1 eV, RF 3000Vpp, Transfer Time 120 μs, Pre Pulse Storage 20 μs, Gas Flow Rate 20%). Spectra were analyzed using the Unidec software^34^.

### Crosslinking mass spectrometry

Wild-type or E351K hMLKL (2.5 μg) were crosslinked in the presence and absence of 500 μM ATP. DSS and BS^3^ were used at 0.1, 0.25, 0.5, 1.0, and 2.0 mM while DMTMM was used at 100, 30, 15, and 7.5 mM. All reactions were incubated at 20 °C for 30 min and stopped by addition of reducing SDS loading dye, before boiling and resolution by 4–20% Tris-Glycine SDS-PAGE (BioRad, CA). Protein bands were visualized with SimplyBlue SafeStain (ThermoFisher) and manually excised for in-gel reduction with 10 mM dithiothreitol (DTT; Sigma) for 30 min, alkylated for 30 min with 50 mM iodo-acetamide (Sigma), and digested with 375 ng trypsin gold (Promega) for 16 h at 37 °C. The extracted peptide solutions were then acidified (0.1% formic acid) and concentrated to 10 μL by centrifugal lyophilization using a SpeedVac AES 1010 (Savant). Extracted peptides were injected and separated by reversed-phase liquid chromatography (LC) on a M-class UHPLC system (Waters, USA) using a 250 mm × 75 μm column (1.7 μm C18, packed emitter tip; Ion Opticks, Australia) with a linear 90-min gradient at a flow rate of 400 nL/min from 98% solvent A (0.1% Formic acid in Milli-Q water) to 35% solvent B (0.1% Formic acid, 99.9% acetonitrile). The nano-UPLC was coupled on-line to a Q-Exactive Orbitrap mass spectrometer equipped with a nano-electron spray ionization source (Thermo Fisher Scientific, Bremen, Germany) or an Impact II UHR-QqTOF (Bruker, Bremen, Germany). High-mass accuracy MS data were obtained in a data-dependent acquisition mode.

Raw files were analyzed using MaxQuant (version 1.5.5.1). The database search was performed using the Uniprot *Homo sapiens* database plus common contaminants with strict trypsin specificity allowing up to two missed cleavages. MaxQuant APL files were converted to MGF files using the APL to MGF convertor software (https://www.wehi.edu.au/people/andrew-webb/1298/apl-mgf-converter). Crosslinked peptides were identified from the MGF files using the StavroX software (version 3.6.0.1). Lysines, protein N-termini, serines, threonines, and tyrosines were set as reaction sites of the crosslinker NHS esters (DSS and BS^3^) or Lysines or protein N-termini linked to aspartic and glutamic acids or protein C-terminus (DMTMM). Trypsin was set as the enzyme allowing for three missed cleavages at lysines and two at arginines. Precursor precision was set at 10 ppm with fragment ion precision set at 20 ppm.

### Hydrogen–deuterium exchange mass spectrometry

Experiments were initiated by 20-fold dilution of protein (1 mg/mL final) in deuterated buffer containing 10 mM Tris-HCl pD 7.5. Aliquots were taken at multiple time points (30 s, 60 s, 5 min, 10 min, 1 h and 16 h) with the hydrogen/deuterium exchange reaction suppressed by acidification of the sample to pH 2.5 using formic acid before snap-freezing in liquid nitrogen. Digestion of the protein was carried out by thawing the sample in a ten-fold dilution of H_2_O before addition of an equimolar concentration of pepsin (Sigma, USA) for 5 min on ice. Peptides were subjected to LC-MS analysis using an 1100 series HPLC (Agilent, USA) coupled to an LTQ-Orbitrap XL (Thermo, USA). Peptides were loaded onto an in-house packed, reverse-phase trap column (ReproSil-Pur C18 (Dr. Maisch GmbH, Germany), 2 × 2 mm^2^, 5 μm) before separation on an in-house packed, reverse-phase analytical column (ReproSil-Pur C18 (Dr. Maisch GmbH), 200 μm×150 mm, 3 μm) housed at 1 °C. Peptides were loaded onto the trap column at 5% acetonitrile and 0.2% formic acid, and elution performed using a gradient rising from 5% to 40% acetonitrile over 12 min, then 85% acetonitrile for 5 min before reconditioning the column at 5% acetonitrile for 15 min. Spectra were acquired in positive ion mode with *m*/*z* range from 350 to 1850. Each sample was analyzed in triplicate. Experiments were performed as experimental replicates (three independent exchange time courses). Deuteration of peptides was determined by analysis of samples using HDX workbench, as previously described^35^. Additional analysis of HDX workbench output was performed using in-house scripts written in R (version 3.3.1). Deuterium exchange per residue was calculated by summing and averaging deuterium exchange across overlapping peptide regions for each residue. The first two residues of each peptide were not included in this calculation due to known issues with rapid back exchange^36^.

### Modeling of the monomeric and tetrameric conformers

An initial monomeric structural model was generated using MODELLER using the PDB entries, 4BTF, 2MSV, and 4MWI, as templates against the hMLKL sequence. Conformational change in this model was predicted using normal mode analysis (NMA) employing the ProDy software^37^. Since large amplitude motions are not harmonic, an eigenvector tracking approach was used to model domain motion and reorientation. Using the X-ray crystal structure, the eigenvector corresponding to the lowest-frequency normal mode was determined using the ProDy NMA application. Atomic coordinates were displaced along this eigenvector, and the geometry minimized using the YASARA software. NMA was performed upon the new geometry, and atomic coordinates again displaced in the direction of the eigenvector with the largest overlap with the original (X-ray structure) lowest-energy eigenvector. This procedure was repeated until the two domains were in contact. To the final structure obtained following NMA tracking, 30 ns of MD simulation was applied using the YASARA software.

The tetramer model generated from SAXS data by SASREF rigid-body fit was subjected to MD refinement using the YASARA software (www.yasara.org). Initial simulated annealing minimization was proceeded by 1 ns of MD using the YASARA2 knowledge-based force field, followed by simulated annealing minimization. The N_ζ_ atoms of lysine residues 157, 173, and 305 were restrained to the same residue in each of the neighboring monomers with a weak harmonic restraint of 10/Nm with a distance of 30 Å. All bonds to hydrogen and angles involving hydrogen were fixed, the simulation temperature was set at 298 K, and the time step used was 2 fs.

### Antibodies and reagents

Primary antibodies used in this study were: rat anti-MLKL (clone 3H1, produced in-house^1^; available as MABC604, EMD Millipore, Billerica, MA, USA; 1:1000), rabbit anti-MLKL phospho-S358 (AB187091, Abcam; 1:4000), anti-GAPDH (2118, Cell Signaling Technology, Danvers, MA, USA; 1:2000), anti-Actin (A-1987, Sigma-Aldrich, St Louis, MO, USA; 1:3000), and anti-VDAC1 (AB10527, EMD Millipore; 1:5000). Recombinant hTNF-Fc, produced in-house, and the Smac mimetic, Compound A, have been previously described^38,39^. The pan-caspase inhibitor, IDN-6556/emricasan, was provided by Tetralogic Pharmaceuticals.

### Generation of *MLKL*^*−/−*^ cell lines and expression constructs

Third-generation lentiviral vectors (pVSVg and pCMV ΔR8.2) were used to generate lentiviral particles in HEK293T cells to stably infect U937 with humanized *Streptococcus pyogenes* cas9 using FUCas9Cherry^40^, which was a gift from Marco Herold. FgH1tUTG, also from Dr Herold, with an inducible sgRNA sequence specifically targeting the first exon of MLKL (TCCCGGAGCTCTCGCTGTTACTTC) was then introduced using an integrase-deficient lentiviral vector, psPAX2-D64V, which was a gift from David Rawlings and Andrew Scharenberg (Addgene plasmid #63586)^41^. For this second infection, virus particles were generated in HEK293T cells seeded into 10 cm plates, co-transfected with these plasmids and a pVSVg helper plasmid. Viral supernatant was harvested at 48 and 72 h postinfection, pooled, and passed through 0.45 μm pore filters, then centrifuged at 20,000 × *g* for 4 h on a sucrose cushion (20% w/v sucrose in 50 mM Tris-HCl, pH 7.4, 100 mM NaCl, 0.5 mM EDTA^42^). Pellets were resuspended in 500 μL RPMI medium (8% fetal calf serum (FCS)), with 4 μg/mL polybrene, and used to infect 5 × 10^5^ cells. Cells were spun at 200 × *g* at 32 °C for 1 h, then incubated overnight at 37 °C. The following day, 1 mL additional media was added, and sgRNA expression was induced using doxycycline (1 μg/mL). Two days after induction, cells were fluorescence-activated cell sorted into pools based on high expression of green fluorescent protein (GFP) to confirm the presence of the guide vector, and after a further week, cells were single-cell sorted based on the absence of GFP to confirm absence of integration. Clones were expanded and treated with TNF (100 ng/mL) and the Smac-mimetic Compound A (500 nM) and the pan-caspase inhibitor IDN-6556 (10 μM) to determine clones no longer responsive to necroptotic stimuli. Effective knockout of MLKL was confirmed using anti-MLKL western blot and Next-Generation sequencing (Illumina).

Mutations were introduced into the hMLKL template (from DNA2.0, CA) using oligonucleotide-directed overlap PCR. Wild-type and MLKL mutant DNA sequences were introduced into the doxycycline-inducible, puromycin-selectable vector, pF TRE3G PGK puro (kindly supplied by Dr Toru Okamoto) as BamHI-EcoRI fragments, as previously described^1,7,11^, and inserts were verified by Sanger sequencing (Micromon DNA Sequencing Facility, VIC, Australia). Vector DNA was co-transfected into HEK293T cells with pVSVg and pCMV ΔR8.2 helper plasmids to generate lentiviral particles as above.

### Cell death assays

The histiocytic lymphoma U937 (sourced from ATCC) and colorectal adenocarcinoma HT29 (a kind gift from Professor Mark Hampton and Dr Andreas Konigstorfer, University of Otago, New Zealand) cell lines were cultured in human tonicity RPMI medium and Dulbecco’s modified Eagle’s medium, respectively, supplemented with 8% v/v FCS, with puromycin (5 μg/mL) added for lines stably expressing MLKL constructs. Routine testing confirmed cell lines to be mycoplasma-negative. U937 cells were plated to 5 × 10^4^ cells/well in 96-well plates and treated with doxycycline (20 ng/mL) overnight to induce the expression of MLKL construct. HT29 cells were assayed analogously, but in 48-well plates with protein expression induced by doxycycline for 4 h before stimulation with necroptotic stimuli. U937 cells were then treated with TNF (100 ng/mL) and the Smac-mimetic Compound A (500 nM) (TS) to induce apoptosis or TS in the presence of the pan-caspase inhibitor IDN-6556 (10 μM) to induce necroptosis for 6, 12, or 24 h; HT29 cells were examined at 48 h post-TSI stimulation. Cells were then compared to untreated cells at the same time point via PI (1 μg/mL) uptake as quantified by flow cytometry.

### Western blot and Blue Native PAGE

Expression analyses were done in parallel with cell death assays, with cells seeded into 96-well plates at 5 × 10^4^ cells/well, and induced overnight with doxycycline, as indicated above, or untreated. When the cell death assay reached 6 and 12 h time points post-TS/TSI treatment, cells for expression analysis were harvested using a 2× SDS Laemmli lysis buffer, sonicated, boiled at 100 °C for 5 min, and then resolved by 4–20% Tris-Glycine gel (Biorad). After transfer onto polyvinylidene difluoride (PVDF), membranes were blocked with 5% skim milk and then probed with antibodies as indicated. For BN-PAGE, wild-type U937 and U937 *MLKL*^−/−^ cells expressing mutant MLKL variants were seeded into 12-well plates at 5 × 10^5^ cells/well 16 h before treatment. Expression of mutant MLKL constructs was induced with 20 ng/mL doxycycline for 4 h, then treated with TSI as described above for time course indicated in Fig. 6 and Supplementary Fig. 8, or left untreated, as indicated. Wild-type U937 were treated with TSI for time course indicated in Fig. 6 or left untreated. For all cell lines, untreated cells were harvested at the longest time point assessed in that experiment. Cells were fractionated into cytoplasmic and membrane fractions as previously described^7,11^. In brief, initial cell permeabilization used a buffer containing 0.025% digitonin (BIOSYNTH, Staad, Switzerland), 2 μM *N*-ethyl maleimide, phosphatase, and protease inhibitors. Crude membrane and cytosolic fractions were separated via centrifugation and then adjusted to a final concentration of 1% w/v digitonin. Fractions were resolved by 4–16% Bis-Tris Native PAGE gel (ThermoFisher) and then transferred onto PVDF for western blot analyses.

### Small-angle X-ray scattering

SAXS data were collected at the Australian Synchrotron SAXS/WAXS beamline using an inline co-flow size-exclusion chromatography set-up^43^. Two-dimensional scattering data were radially averaged, normalized to sample transmission, and 5 × 2 s scatter patterns from the apex of the elution peak were averaged and background scatter (an average of 20 scatter patterns from early in the SEC run) was subtracted using the ScatterBrain software (Stephen Mudie, Australian Synchrotron). The ATSAS suite of software was used for Guinier analysis (PRIMUS^44^), to calculate the pair-wise intra-atomic distance distribution function *P*(*r*) and maximum particle dimension, *D*_max_ (GNOM^45^), and rigid body modeling of the tetramer using SASREF with P4 symmetry^46^. All structural models were illustrated using PyMOL. The data collection and processing statistics are presented in Supplementary Table 1.

### Analytical ultracentrifugation

Sedimentation velocity experiments were performed using an XL-I analytical ultracentrifuge (Beckman Coulter) equipped with UV/Vis scanning optics, as before^22^. Buffer reference (20 mM HEPES pH 7.5, 200 mM NaCl) and 380 μL of hMLKL sample solutions at 0.25, 0.5, or 1 mg/mL were loaded into 12 mm double-sector cells with quartz windows and the cells were mounted in an An-60Ti 4-hole rotor or An-50Ti 8-hole rotor. Experiments in the presence or absence of 300 μM ATP were performed on 1 mg/mL hMLKL samples and buffer reference containing 300 μM ATP was used. All experiments were conducted at 50,000 rpm (201,600 × *g*) and 20 °C, and radial absorbance data were collected at 280 nm in continuous mode. Data were fitted to a continuous sedimentation coefficient distribution [*c*(*s*)] model and converted to standardized sedimentation coefficient (*s*_20,w_) distributions using SEDFIT^47^. The protein partial-specific volumes, buffer density, and buffer viscosity were calculated using SEDNTERP^48^.

### Thermal shift assays

Thermal shift assays were performed as described previously^1,14,16^ using a Corbett Real Time PCR machine with proteins diluted in 150 mM NaCl, 20 mM Tris pH 8.0, 1 mM DTT to 0.5 μg in a total reaction volume of 25 μL. SYPRO Orange (Molecular Probes, CA) was used as a probe with fluorescence detected at 530 nm. Thermal stability was examined in the presence and absence of 0.2 mM ATP, with no divalent cations added. A positive Δ*T*_m_ value indicated ATP binding that protected the protein from denaturation. The *K*_d_ for ATP binding was determined by titrating ATP concentrations 0–400 mM. Two transitions were observed for some variants and the *K*_d_ was calculated based on the Δ*T*_m_ of the first transition state. Two independent assays were performed for wild-type and mutant hMLKL proteins; averaged data ± SD are shown for each in Supplementary Fig. 4 and *K*_d_ estimates in Table 1.

### Surface plasmon resonance

SBP-tagged hRIPK3 kinase domain (residues 2–356) was immobilized onto a dextran chip with preimmobilized streptavidin (Biacore SA chip). Briefly, the surface was activated with 50 mM NaOH + 1 M NaCl solution and SBP-hRIPK3 injected at a concentration of 0.04 mg/mL in 20 mM HEPES buffer (pH 8.0) containing 200 mM NaCl. Data were collected using a Biacore 4000 or Biacore T200 (Biacore International, Uppsala, Sweden). As a control, the first flow cell was used as reference surface to correct for bulk refractive index, matrix effects, and nonspecific binding. The second flow cell was immobilized with SBP-hRIPK3. Solutions of full-length wild-type hMLKL and T357E/S358E hMLKL were flowed over the chip at 1, 0.5, 0.25, 0.125, 0.0625, and 0 μM in triplicates, at 30 μL/min, and the amount of bound material as a function of time was recorded as sensorgrams. The dissociation phase was monitored in 20 mM HEPES (pH 8.0) containing 200 mM NaCl at the same flow rate. This was followed by one injection of 50 mM NaOH + 1 M NaCl to regenerate a fully active capturing surface. All experiments were performed at 25 °C.

### Data availability

Atomic coordinates for the T357E/S358E hMLKL (190–471) have been deposited in the Protein Data Bank under the accession number 6BWK. The data that support the findings of this study are available from the corresponding author upon request.

## Electronic supplementary material

Supplementary Information

Description of Additional Supplementary Files

Supplementary Data 1
