# Supplementary material for: Conformational switching of the pseudokinase domain promotes human MLKL tetramerization and cell death by necroptosis
Source: Nat Commun. 2018 Jun 21;9:2422. doi: 10.1038/s41467-018-04714-7 (PMC6013482; doi:10.1038/s41467-018-04714-7)
Supplement: Supplementary file 1 — Supplementary Information [file 41467_2018_4714_MOESM1_ESM.pdf]

Supplementary Figure 1

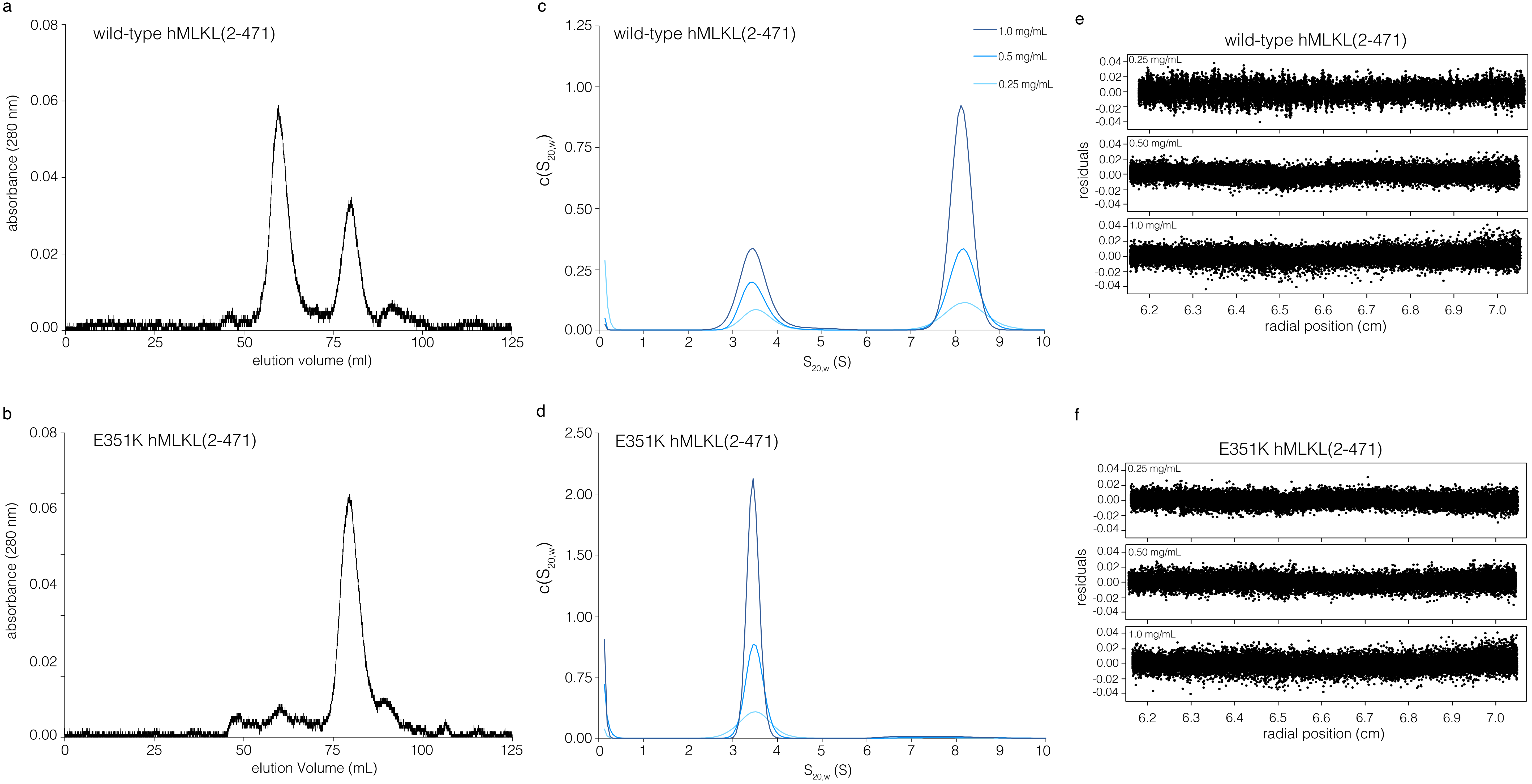

Wild-type hMLKL exists in a monomer-oligomer equilibrium; E351K hMLKL is monomeric.

Size Exclusion Chromatography profiles of (a) wild-type hMLKL and (b) E351K hMLKL. Approximately 1 mg of protein was loaded onto S200 16/600 column (GE Healthcare) at a flow rate of 1 mL/min was eluted in 20 mM HEPES pH 7.5, 200 mM NaCl, 5% glycerol. Standardized continuous sedimentation coefficient [c(s20,w)] distributions for (c) wild-type hMLKL(2-471) and (d) E351K MLKL(2-471) at 1.0, 0.5 and 0.25 mg/mL. Residuals for the best fit of the raw radial absorbance sedimentation velocity data to a continuous sedimentation coefficient [c(s)] distribution model for wild-type (e) and E351K (f) hMLKL(2-471) at 0.25 mg/mL (top), 0.5 mg/mL (middle) and 1.0 mg/mL (bottom).

Supplementary Figure 2

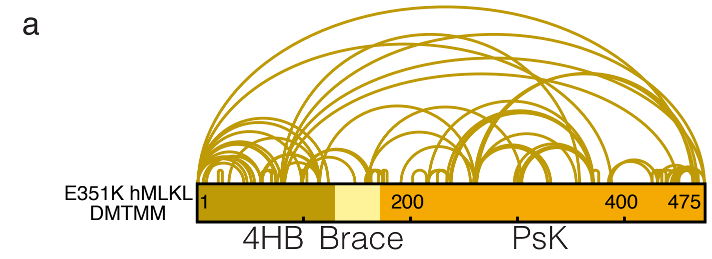

b

Residue	Best Score	Minimum Distance (Å)	Cross-linker
Lysine 157	145	29.4	BS3
Lysine 173	167	29.8	BS3
Lysine 173	161	29.8	DSS
Lysine 305	179	29.5	DSS
Lysine 305	167	29.5	BS3

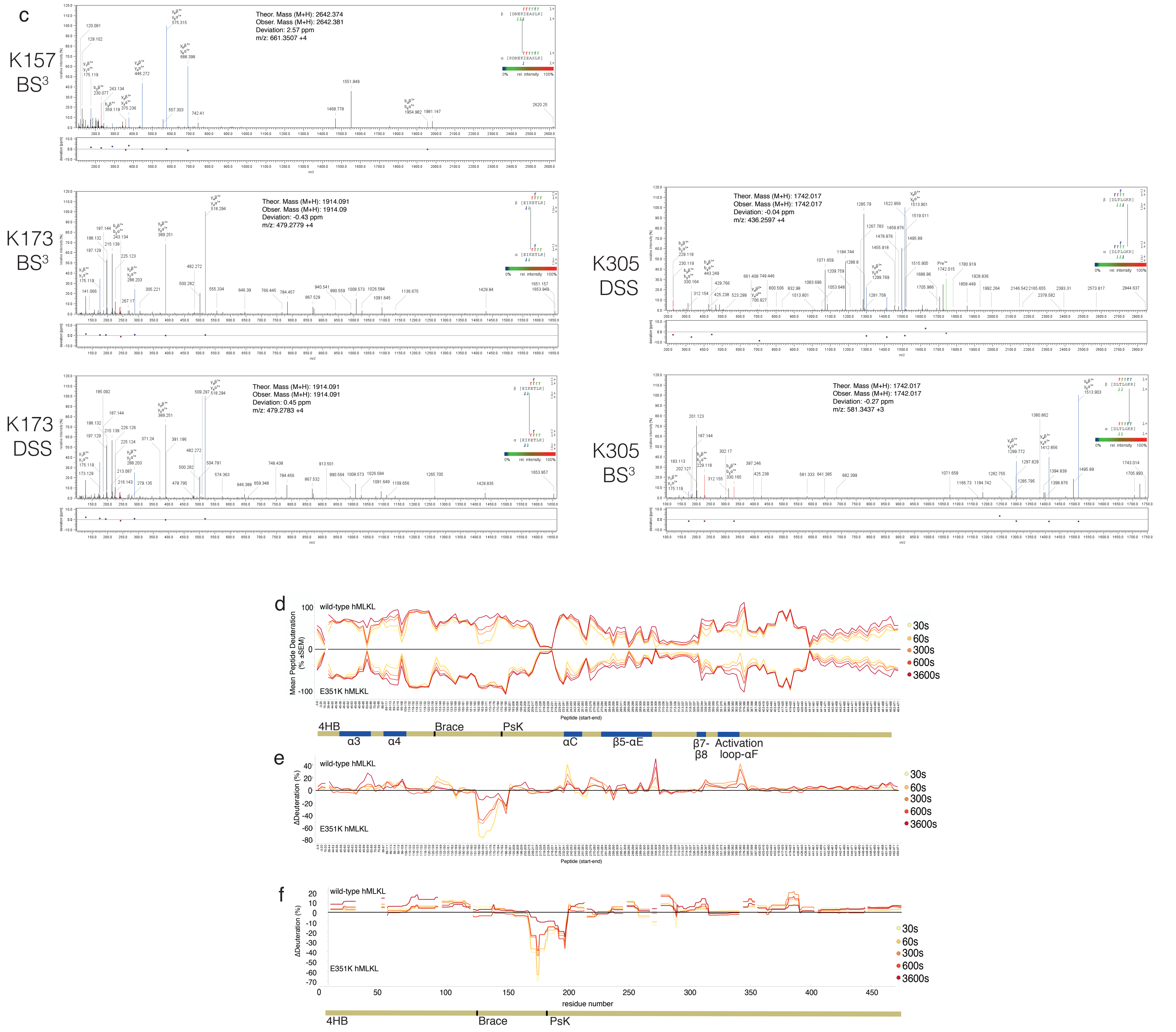

Summary of DSS/BS³ hMLKL inter-subunit crosslinks and monomer vs tetramer HDX.

(a) Map of intramolecular DMTMM crosslinks observed in E351K hMLKL, as listed in Supplementary Table 1. (b) The best scoring inter-subunit crosslinks from DSS and BS³ coupled hMLKL. A complete list of crosslinks is shown in Supplementary Table 1. (c) Annotated MS/MS spectra of the best scoring inter-subunit crosslinks from DSS and BS³ coupled hMLKL.
(d) Mirror plot demonstrating deuteration (percentage of theoretical maximum) of wild-type vs E351K hMLKL peptides over the indicated time points. All SEM values were below ±7.86 from n=3; error bars omitted for clarity.
(e) Differential deuterium uptake of wild-type vs E351K hMLKL peptides over the indicated time points. Schematic of MLKL architecture is shown between panels (d) and (e).
(f) Differential deuterium uptake of wild-type vs E351K hMLKL for each analyzed peptide was averaged across overlapping sequences per amino acid residue (excluding the first 3 residues of each peptide due to high rates of back exchange). Gaps in lines represent regions of the protein without sufficient sequence coverage. Schematic of the corresponding MLKL domains is shown at the foot of the panel.

Supplementary Figure 3

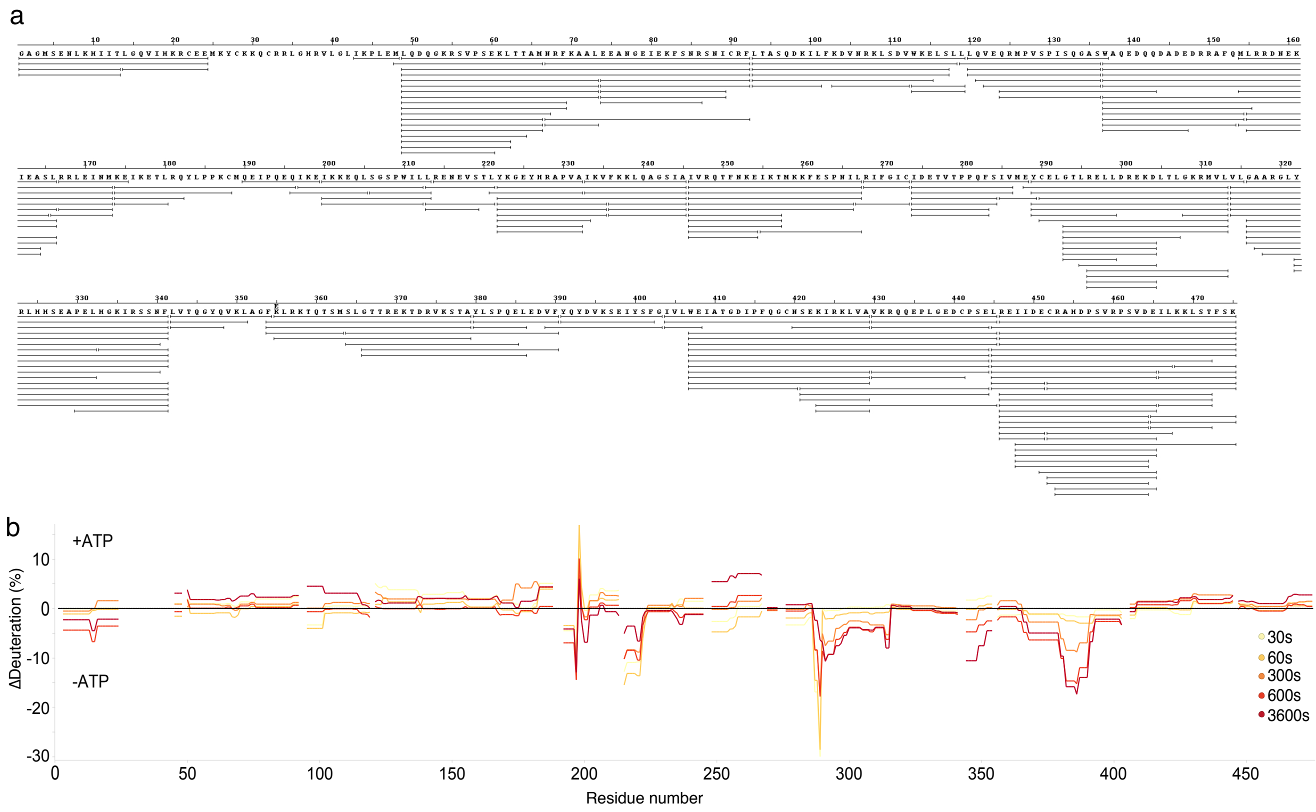

Summary of HDX sequence coverage and E351K hMLKL HDX in the presence or absence of ATP.

(a) Summary of sequence coverage and peptides used for hydrogen-deuterium exchange experiments.
(b) Differential deuterium uptake of E351K hMLKL +ATP vs -ATP for each analyzed peptide was averaged across overlapping sequences per amino acid residue (excluding the first 2 residues of each peptide due to high rates of back exchange). Gaps in lines represent regions of the protein excluded from analysis owing to insufficient sequence coverage.

Supplementary Figure 4

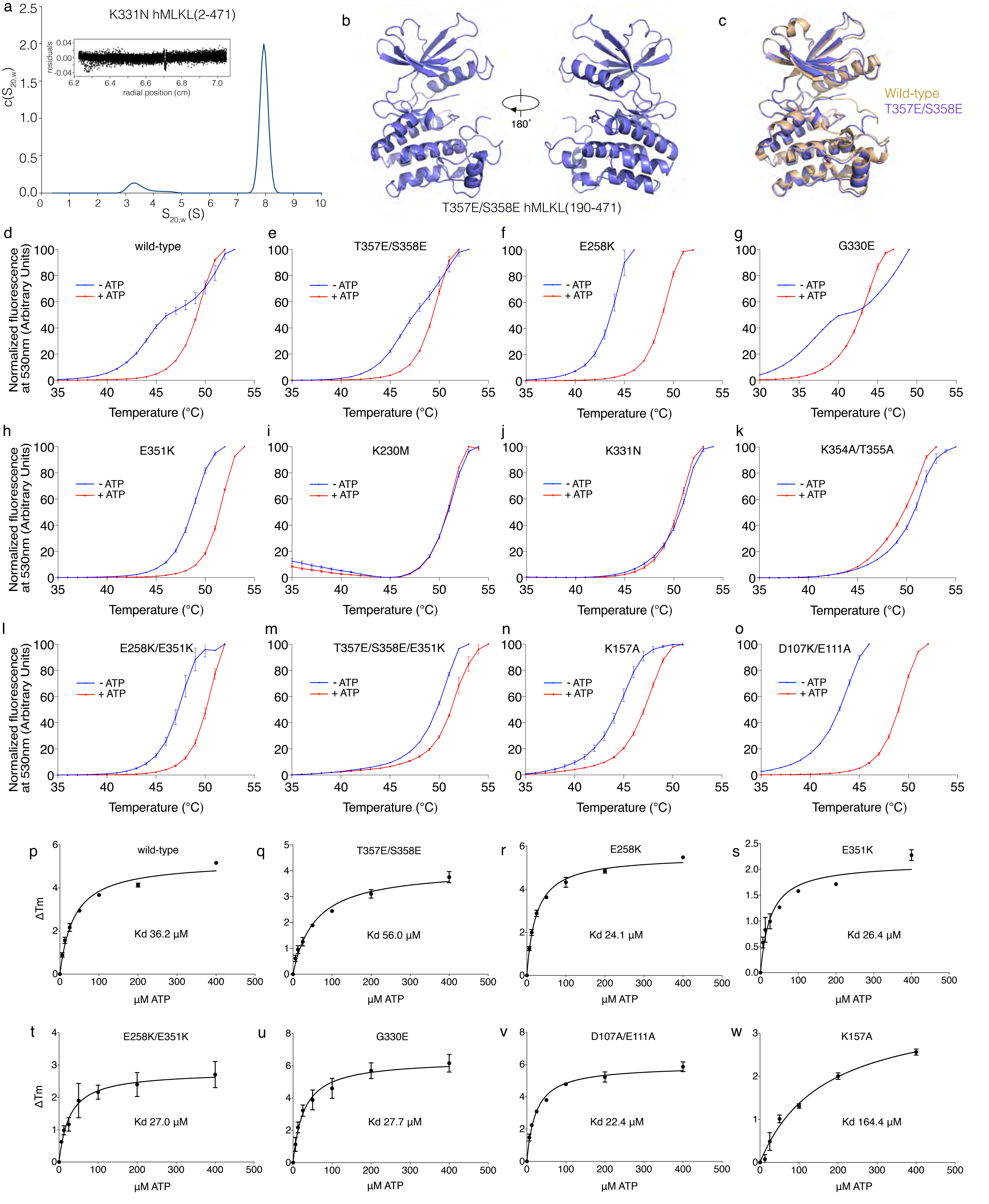

Biophysical characterization of hMLKL mutants by AUC, X-ray crystallography and thermal shift assay

(a) Recombinant K331N hMLKL(2-471) exists principally as a tetramer in solution in AUC studies.
(b) Crystal structure of T357E/S358E hMLKL PsK domain does not reveal major structural perturbations arising from mutation.
(c) Overlay of T357E/S358E hMLKL PsK (PDB 6BWK) and wild-type hMLKL PsK domain structure (PDB 4MWI).
(d-o) Thermal denaturation of wild-type and mutant hMLKL(2-471) proteins in the absence (blue) and presence (red) of 400 μM ATP. Mean±SEM shown for ≥2 experiments for each data point.
(p-w) Binding isotherms illustrating the thermal shift in the presence of ATP relative to apo hMLKL (ΔTm) vs ATP concentration used to determine K_d for ATP binding. Data are mean±SEM for ≥2 experiments.

Supplementary Figure 5

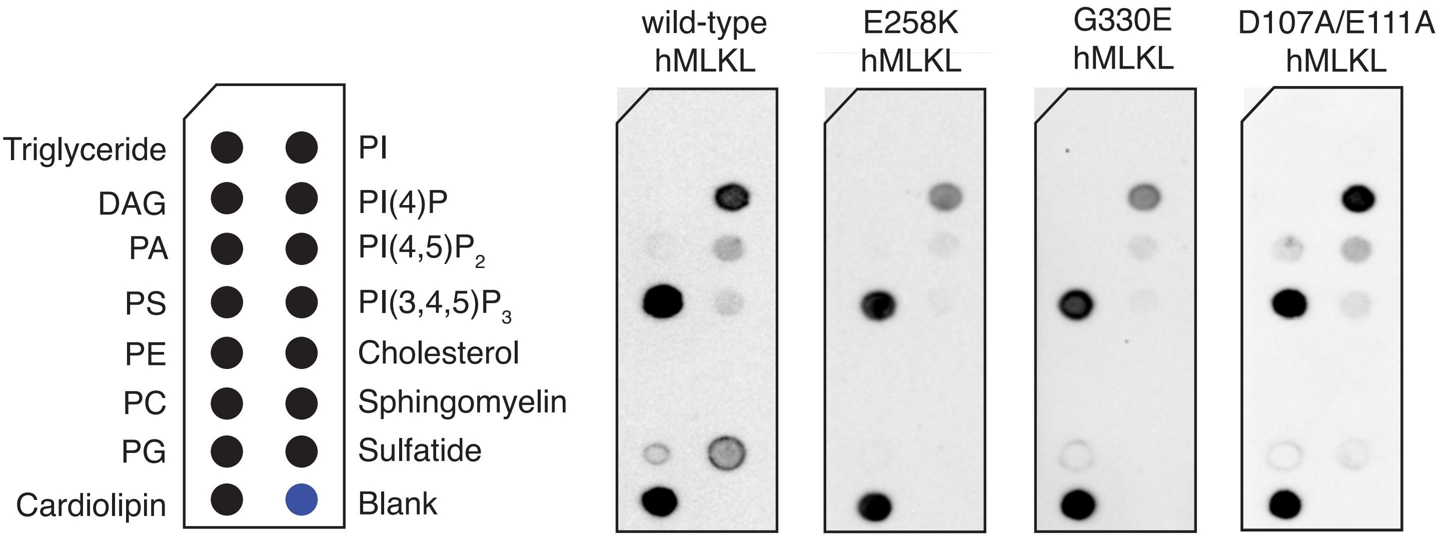

Wild-type, E258K, G330E and D107A/E111A hMLKL bind membrane lipids.

200 ng/mL recombinant full length hMLKL were incubated with membrane lipid arrays (Echelon Biosciences) and binding evaluated by western blotting using an anti-MLKL (3H1 clone) antibody. Wild-type and mutant hMLKL exhibited preferences for phosphatidylinositol (4)-phosphate (PI(4)P), cardiolipin and phosphatidylserine (PS), with some binding to phosphatidylinositol (4,5)-biphosphate (PI(4,5)P_2) and phosphatidylinositol (3,4,5)-phosphate (PI(3,4,5)P_3) observed. PI, phosphatidylinositol; PE, phosphatidylethanolamine; PA, phosphatidic acid; DAG, diacylglyceride; PC, phosphatidylcholine; PG, phosphatidylglycerol; Sulfatide, 3-sulfogalactosylceramide. A representative of duplicate experiments is shown.

Supplementary Figure 6

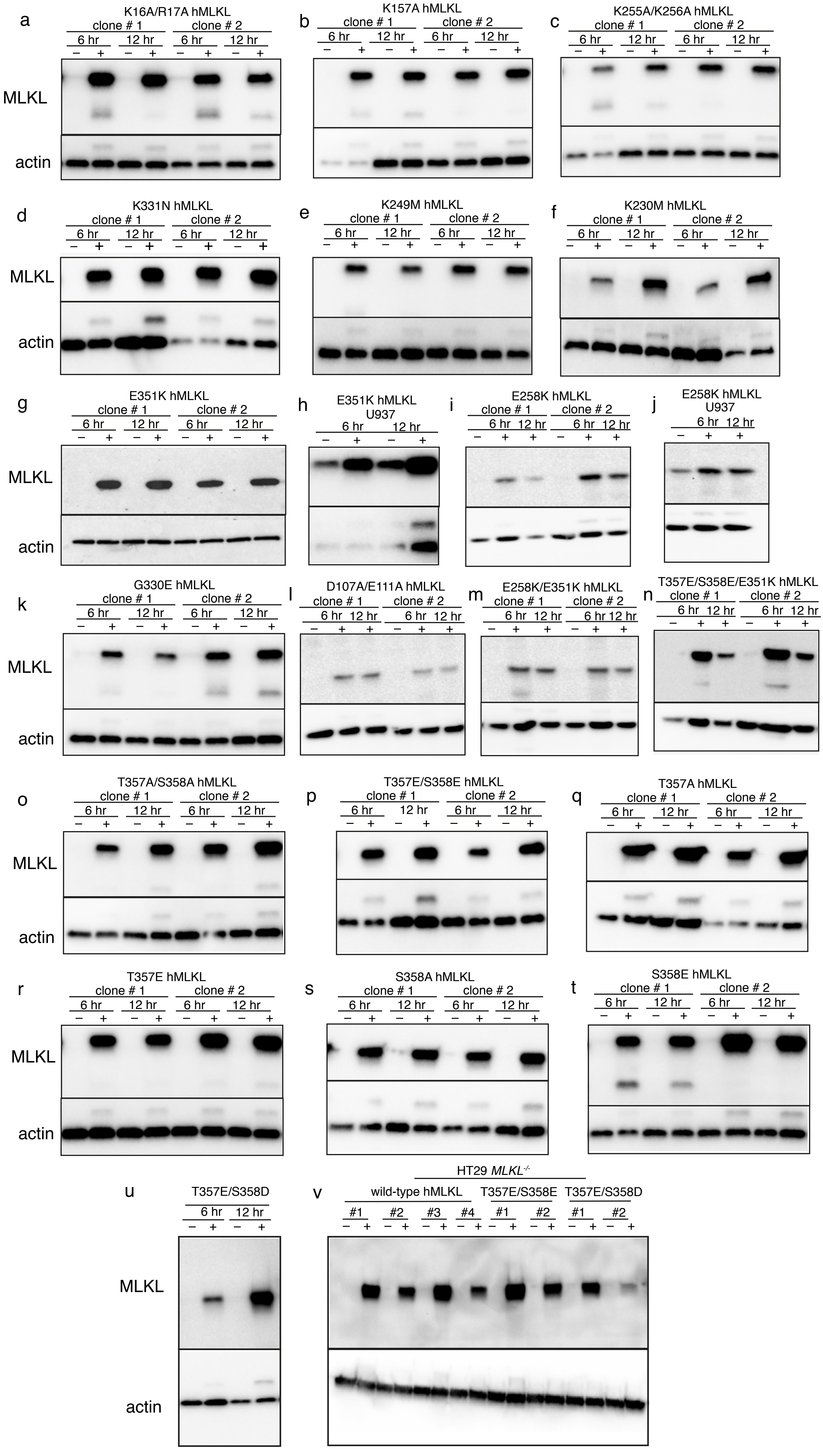

Western blot validation of hMLKL expression in wild-type or *MLKL$^{-/-}$* U937 cells.

Lysates of cells uninduced or induced with doxycycline were resolved by reducing SDS-PAGE and transferred to PVDF membrane for evaluation of MLKL expression levels by western blot using 3H1 rat monoclonal antibody. Expression of doxycycline-inducible constructs of wild-type and mutant hMLKL was evaluated in two independent *MLKL$^{-/-}$* U937 clones: (a) K16A/R17A; (b) K157A; (c) K255A/K256A; (d) K331N; (e) K249M; (f) K230M; (g) E351K; (i) E258K; (k) G330E; (l) D107A/E111A; (m) E258K/E351K; (n) T357E/S358E/E351K; (o) T357A/S358A; (p) T357E/S358E; (q) T357A; (r) T357E; (s) S358A; (t) S358E; and one clone for T357E/S358D (u). Expression of E351K (h) and E258K (j) in stably-transfected parental U937 cells was also assessed.
(v) Expression of doxycycline-inducible constructs of wild-type hMLKL (4 clones), T357E/S358E and T357E/S358D mutant hMLKL (2 clones each) was evaluated in edited *MLKL$^{-/-}$* HT29 cells at 4 h post induction. An anti-actin reprobe of the blots was performed as a loading control.

Supplementary Figure 7

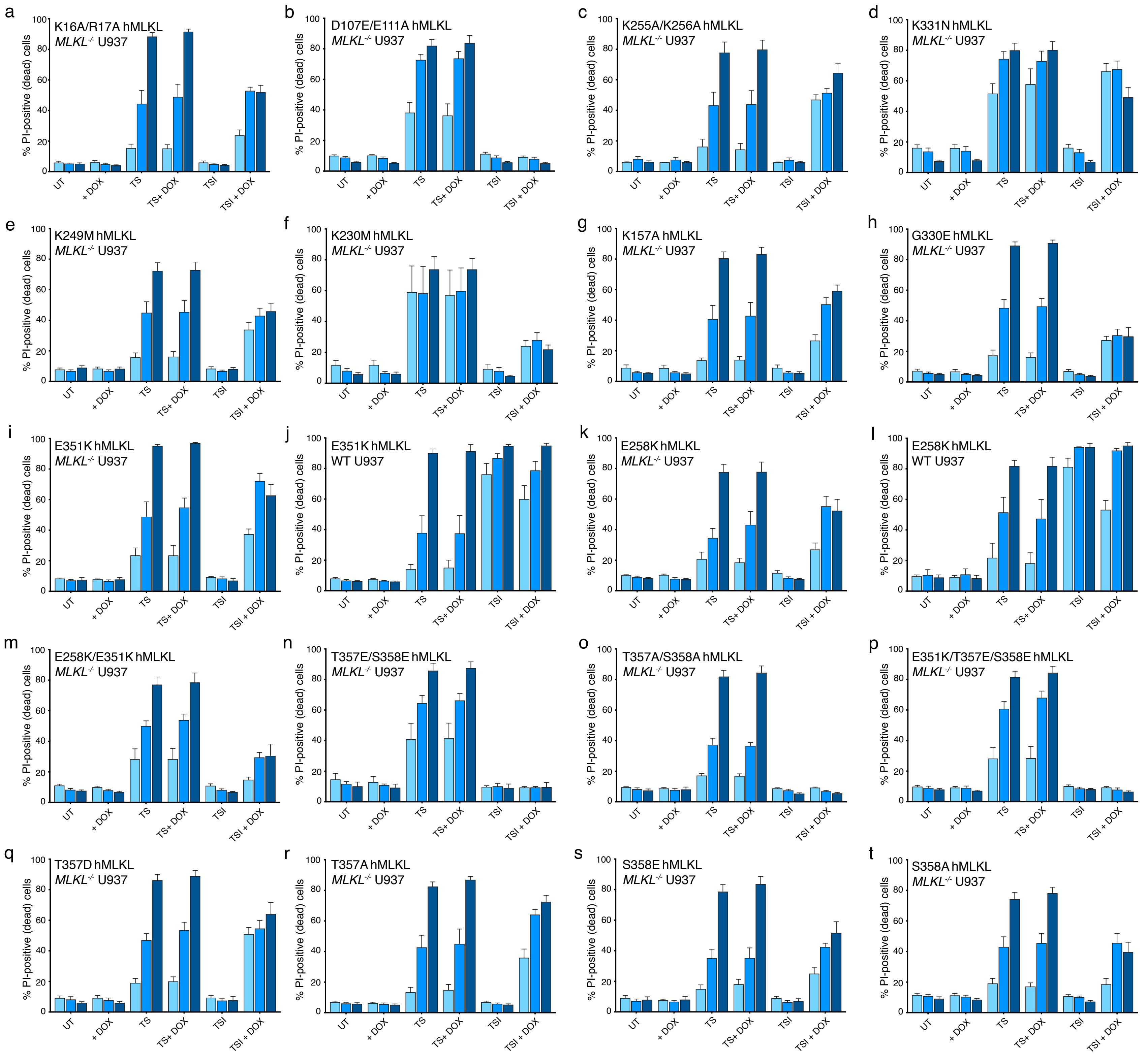

Cell death time course of *MLKL*$^{-/-}$ U937 cells expressing hMLKL constructs following treatment with various stimuli.

Cell death was monitored by flow cytometry, using PI uptake, at 6, 12 and 24 hours after treatment with TS (to induce apoptosis) or TSI (to stimulate necroptosis) in the presence and absence of doxycycline-induced hMLKL exogene expression. Data represent at least 3 independent assays conducted in 2 independent cell lines as mean ± SEM. Cell death was evaluated for *MLKL*$^{-/-}$ U937 cells harboring doxycycline-inducible hMLKL mutants: (a) K16A/R17A; (b) D107A/E111A; (c) K255A/K256A; (d) K331N; (e) K249M; (f) K230M; (g) K157A; (h) G330E; (i) E351K; (k) E258K; (m) E258K/E351K; (n) T357E/S358E; (o) T357A/S358A; (p) E351K/T357E/S358E; (q) T357D; (r) T357A; (s) S358E; (t) S358A. Single parental U937 lines harboring the hMLKL mutants, E351K (j) and E258K (l), were also evaluated.

Supplementary Figure 8

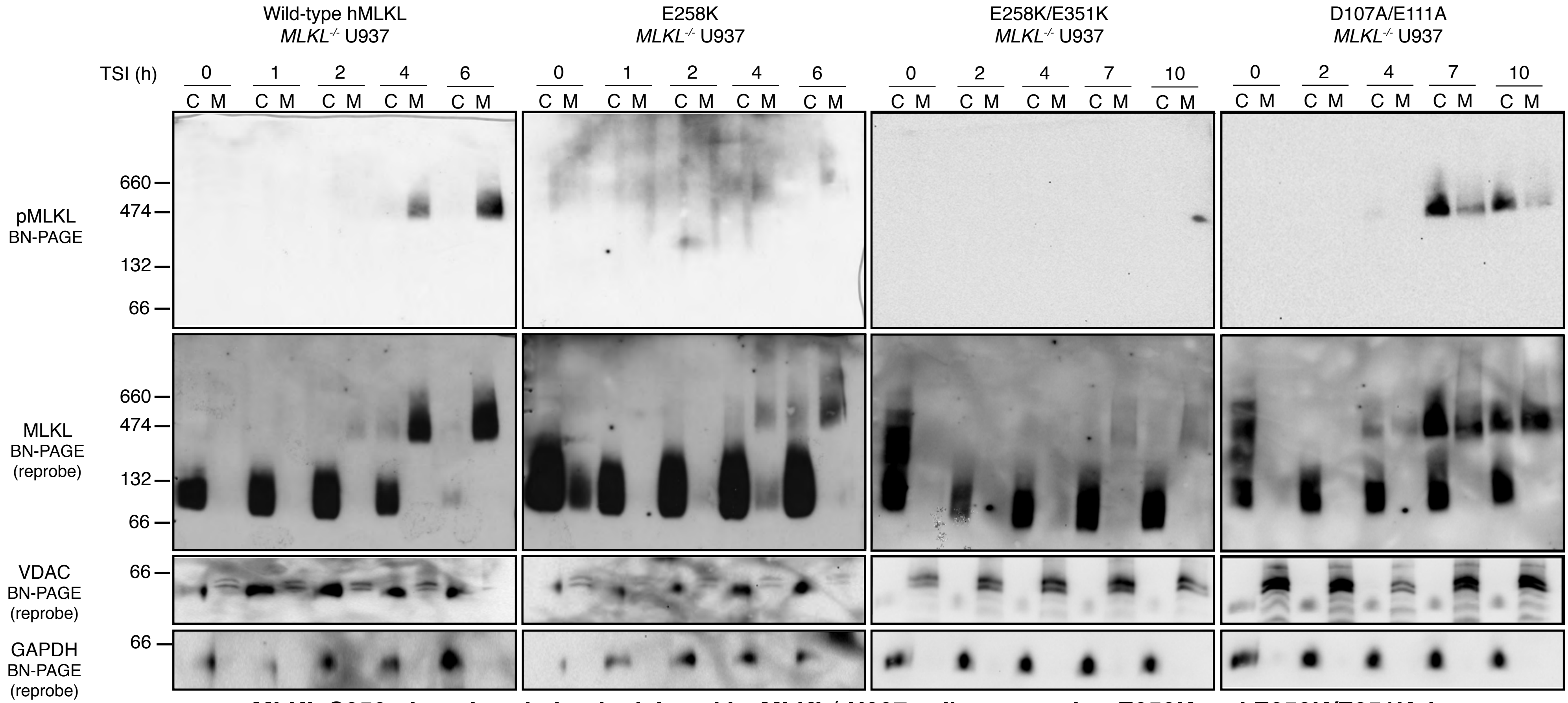

MLKL S358 phosphorylation is delayed in *MLKL*$^{-/-}$ U937 cells expressing E258K and E258K/E351K, but not D107A/E111A, hMLKL constructs following treatment with necroptotic (TSI) stimuli.

MLKL$^{-/-}$ edited U937 cells reconstituted with wild-type hMLKL or the E258K, E258K/E351K or D107A/E111A mutants were assessed for MLKL S358 phosphorylation and oligomer formation by Blue-Native PAGE post-TSI treatment at indicated time points.
Separation into cytoplasmic (C) and membrane (M) fractions was validated by reprobes for GAPDH (cytoplasmic) then VDAC1 (membrane; seen as a doublet). All blots are representative of ≥2 independent experiments and phosphorylation was consistently detected in wild-type, E258K and D107A/E111A late time points. Laddering in lane 1 of E258K/E351K and D107A/E111A gels arises from cross-reactivity of western blot antibodies with the molecular weight marker.

Supplementary Table 1. Statistics for SAXS data collection.

**Statistics for SAXS data collection on the K331N hMLKL(2-471) tetramer**	
Instrument	Australian Synchrotron SAXS/WAXS beamline[1]
Beam geometry	120 μm point source
Wavelength (Å)	1.033
Exposure time	2 sec exposures
Temperature (K)	285
q range ($Å^{-1}$)[#]	0.0060 to 0.25
Protein concentration	50 μL of 7.8 mg/ml protein *via* inline gel filtration chromatography in 0.2M NaCl, 20mM HEPES pH 7.5
**Structural parameters**	
I(0) (cm^{-1}) [from P(r)]	0.0652 ± 0.0001
R_g (Å) [from P(r)]	52.29 ± 0.01
D_{max} (Å)	170
I(0) (cm^{-1}) (from Guinier)	0.0651 ± 0.0003
R_g (Å) (from Guinier)	52.20 ± 0.43
**Software employed**	
Primary data reduction	Scatterbrain (Australian Synchrotron)
Data processing	PRIMUS[2], GNOM[3]
Computation of model intensities	CRYSOL[4]
Rigid body fitting	SASREF[5]

[#] q is the magnitude of the scattering vector, which is related to the scattering angle (2θ) and the wavelength (λ) as follows: $q = (4\pi/\lambda)\sin\theta$

Supplementary Table 2. Data collection and refinement statistics (molecular replacement)

	T357E/S358E hMLKL(190-471)
**Data collection**	
Space group	C 2 2 2_1
Cell dimensions	
a, *b*, *c* (Å)	72.114 74.723 127.601
α, β, γ (°)	90, 90, 90
Resolution (Å)	40.26 - 2.79 (2.89 - 2.79)*
R_{merge}	0.215 (4.17)
$I / \sigma I$	7.66 (0.44)
Completeness (%)	0.97 (0.83)
Redundancy	6.2 (5.6)
**Refinement**	
Resolution (Å)	40.25 - 2.79 (3.19 – 2.79)
No. reflections	53075 (6400)
R_{work} / R_{free}	0.2567/0.3032
No. atoms	
Protein	1963
Ligand/ion	22
Water	0
B-factors	
Protein	88.40
Ligand/ion	95.66
Water	N/A
R.m.s. deviations	
Bond lengths (Å)	0.004
Bond angles (°)	0.66

*Values in parentheses are for highest-resolution shell.
Number of crystals used for structure = 1.

SUPPLEMENTARY METHODS

Crystallization of T357E/S358E hMLKL(190-471)

T357E/S358E mutation was introduced into the transcript encoding hMLKL (190-471) by oligonucleotide-directed PCR and cloned as a BamHI-EcoRI fragment into pFastBac Htb (Invitrogen) for bacmid generation, baculovirus preparation and expression in Sf21 insect cells, essentially as reported for Wt and other mutants previously[7]. Following TEV protease cleavage, a vector encoded overhang remains in hMLKL (190-471): GAMGS. Purification was performed as described previously [7]. Briefly, Sf21 cell pellets from a 0.5L expression were resuspended in 0.5 M NaCl, 20 mM Tris pH 8.0, 10 mM imidazole pH 8.0, 20% v/v glycerol containing 0.5 mM TCEP, EDTA-free Complete Protease Inhibitor (Roche) and 2 mM PMSF, and lysed by sonication before debris was eliminated by centrifugation at 45 000 x g, 4°C, 30 min. Supernatant was mixed with HisTag Ni resin at 4°C, 60 min, before beads were washed with 0.5 M NaCl, 20 mM Tris pH 8.0, 10 mM imidazole pH 8.0, 20% v/v glycerol containing 0.5 mM TCEP, and a comparable buffer containing 35mM imidazole pH 8.0. Protein was eluted in a comparable buffer containing 250 mM imidazole pH 8.0. The His-tag proteolytically-cleaved by TEV protease (200 μg) at 20°C for 2 h, before dialyzing extensively against 200 mM, 20 mM HEPES pH 7.5, 5% v/v glycerol in 12-14 kDa MWCO tubing. Dialysate was recovered, insoluble material pelleted by centrifugation and supernatant applied to a 1mL HisTrap Ni column (GE Healthcare) by peristaltic pump. The protein eluted in the unbound fraction with further washes in 200 mM NaCl, 20 mM HEPES pH 7.5, 5 mM imidazole pH 8.0, 5% v/v glycerol, and was concentrated by centrifugal ultrafiltration (10kD MWCO, Sartorius) for application to Superdex 200 10/300 column (GE Healthcare). Protein eluted as a single peak corresponding to a monomer in 200mM NaCl, 20 mM HEPES pH 7.5, 5% v/v glycerol, and purity assessed by reducing SDS-PAGE with SimplyBlue SafeStain (Thermo Fisher) staining. Pure fractions were pooled and concentrated by centrifugal ultrafiltration to ~10 mg/mL. Aliquots were snap frozen in liquid nitrogen and stored at –80°C until required.

Crystallization of T357E/S358E hMLKL(190-471)

A sparce matrix screen was set up 20°C at the C3 Facility (Parkville, VIC) with 1:1 sitting drops of 150 nL 5mg/mL protein:150 nL reservoir solution. After 3 days, crystals were identified in the condition, 5% w/v polyethylene glycol 8000, 20% v/v polyethylene glycol 300, 0.1M Tris

chloride pH 8.5, 10% v/v glycerol. Crystals were harvested from microwell plates and snap cooled in liquid nitrogen using the reservoir buffer as a cryosolution.

Data collection and structure determination

X-ray diffraction data of T357E/S358E hMLKL(190-471) crystals were collected at the Australian Synchrotron MX2 beamline at 100K at a wavelength of 0.9537 Å. Data were processed with XDS[8] to 2.8 Å based on CC1/2[6] and the structure was solved by molecular replacement in PHASER[9] using the wild type human MLKL pseudokinase domain structure (PDB code 4MWI) as a search model. The structure was refined with iterative rounds of building in COOT[10] and refinement in PHENIX[11]. Ramachandran statistics for final model - favoured 94.59%, accepted 5.41%, outliers 0%. Summary statistics are shown in **Supplementary Table 2**.

Membrane lipid arrays

Membrane lipids arrays were essentially performed as described previously[12]. Briefly, membranes (Echelon Biosciences; catalog P-6002) were blocked with fatty acid-free BSA (3% w/v) in TBS-T overnight at 4°C. 200ng/mL recombinant wild-type, E258K, G330E and D107A/E111A hMLKL in 3% (w/v) BSA/TBS-T were incubated with rocking for 1 hour at 20°C and washed extensively with TBS-T. Membranes were incubated with rat anti-MLKL (3H1) in 3% (w/v) BSA/TBS-T for 1 hour at 20°C before extensive washing with TBS-T. Following incubation with anti-rat HRP in 3% (w/v) BSA/TBS-T for 1 hour at 20°C and extensive washing, bound MLKL was visualised by enhanced chemiluminescence.

SUPPLEMENTAL REFERENCES

1. Kirby N, *et al*. Improved radiation dose efficiency in solution SAXS using a sheath flow sample environment. *Acta Crystallogr D Struct Biol* **72**, 1254-1266 (2016).

2. Konarev PV, Volkov VV, Sokolova AV, Koch MHJ, Svergun DI. PRIMUS: a Windows PC-based system for small-angle scattering data analysis. *J Appl Crystallogr* **36**, 1277-1282 (2003).

3. Svergun DI. Determination of the Regularization Parameter in Indirect-Transform Methods Using Perceptual Criteria. *J Appl Crystallogr* **25**, 495-503 (1992).

4. Svergun DI, Barberato C, Koch MHJ. CRYSOL - a Program to Evaluate X-ray Solution Scattering of Biological Macromolecules from Atomic Coordinates. *J Appl Cryst* **28**, 768-773 (1995).

5. Petoukhov MV, Svergun DI. Global rigid body modeling of macromolecular complexes against small-angle scattering data. *Biophys J* **89**, 1237-1250 (2005).

6. Karplus PA, Diederichs K. Linking crystallographic model and data quality. *Science* **336**, 1030-1033 (2012).

7. Murphy JM*, et al*. Insights into the evolution of divergent nucleotide-binding mechanisms among pseudokinases revealed by crystal structures of human and mouse MLKL. *The Biochemical journal* **457**, 369-377 (2014).

8. Kabsch W. Integration, scaling, space-group assignment and post-refinement. *Acta Crystallogr D Biol Crystallogr* **66**, 133-144 (2010).

9. McCoy AJ, Grosse-Kunstleve RW, Adams PD, Winn MD, Storoni LC, Read RJ. Phaser crystallographic software. *J Appl Crystallogr* **40**, 658-674 (2007).

10. Emsley P, Lohkamp B, Scott WG, Cowtan K. Features and development of Coot. *Acta Crystallogr D Biol Crystallogr* **66**, 486-501 (2010).

11. Adams PD*, et al*. PHENIX: a comprehensive Python-based system for macromolecular structure solution. *Acta Crystallogr D Biol Crystallogr* **66**, 213-221 (2010).

12. Wang H*, et al*. Mixed Lineage Kinase Domain-like Protein MLKL Causes Necrotic Membrane Disruption upon Phosphorylation by RIP3. *Mol Cell* **54**, 133-146 (2014).
