# Supplementary material for: Conformational switching of the pseudokinase domain promotes human MLKL tetramerization and cell death by necroptosis
Source: Nat Commun. 2018 Jun 21;9:2422. doi: 10.1038/s41467-018-04714-7 (PMC6013482; doi:10.1038/s41467-018-04714-7)
Supplement: Supplementary file 2 — Description of Additional Supplementary Files [file 41467_2018_4714_MOESM2_ESM.pdf]

Descriptions of Additional Supplementary Files

File Name: Supplementary Dataset 1
Description: Crosslinking-mass spectrometry data and calculated Calpha-Calpha distances in monomer model
